# Ferroptosis regulation by traditional chinese medicine for ischemic stroke intervention based on network pharmacology and data mining

**DOI:** 10.1371/journal.pone.0321751

**Published:** 2025-04-16

**Authors:** Jun Lei, Wei Chen, Yaodong Gu, Xueyan Lv, Xingyu Kang, Xicheng Jiang

**Affiliations:** Heilongjiang University of Chinese Medicine, Harbin, China; Fujian Provincial Hospital, CHINA

## Abstract

**Objective:**

The aim of this study is to use network pharmacology and data mining to explore the role of traditional Chinese medicine (TCM) in ischemic stroke (IS) intervention by ferroptosis regulation. The results will provide reference for related research on ferroptosis in IS.

**Methods:**

The ferroptosis-related targets were obtained from the GeneCards, GeneCLiP3, and FerrDdb databases, while the IS targets were sourced from the GeneCards and DisGeNET databases. Venny was used to identify IS targets associated with ferroptosis. A protein-protein interaction (PPI) analysis was then conducted, and machine learning screening was used to validate these potential targets. The potential targets that met specific criteria and their related compounds allowed us to select TCMs. A mechanistic analysis of the potential targets was conducted using the DAVID database. PPI network diagrams, target-compound network diagrams, and target-compound-TCM network diagrams were then constructed. Finally, molecular docking technology was used to verify the binding activities of the TCM compounds and core components with the identified targets. In addition, the properties, flavors, meridian tropism, and therapeutic effects of the candidate TCMs were analyzed and statistically evaluated.

**Results:**

A total of 706 targets associated with ferroptosis in IS were obtained, and 14 potential ferroptosis targets in IS were obtained using machine learning. Furthermore, 413 compounds and 301 TCMs were screened, and the binding activities of the targets to the TCM compounds and the core prescriptions were stable. The candidate TCMs primarily exhibited cold, warm, bitter taste, pungent taste, liver meridian, heat-cleaning medicinal, and tonify deficiency properties.

**Conclusions:**

This study investigated ferroptosis regulation for IS intervention using TCM. We began by investigating the targets of IS and ferroptosis, and we also analyzed the relevant mechanism of ferroptosis in IS. The results of this study provide reference for related research on ferroptosis in IS.

## Introduction

Stroke is an acute cerebrovascular disorder characterized by damaged brain tissue and impaired brain function. The types of stroke include ischemic stroke (IS) and hemorrhagic stroke [1]. The incidence rate of IS accounts for 87% of all types of stroke, and due to its high mortality and high disability rate, it severely threatens the life and health of residents worldwide. Currently, intravenous thrombolysis and endovascular treatment are the primary IS treatment approaches, with the early rescue of the ischemic penumbra zone and the restoration of its blood supply being of utmost importance for reducing neuronal damage [2]. However, after cerebral blood flow is restored, factors such as inflammatory cytokines, reactive oxygen species, and amino acid toxicity can damage neurons and further aggravate brain tissue injury. The effective reduction of brain tissue damage caused by IS remains a current research focus and challenge.

Ferroptosis is a mode of programmed cell death, and it is a process where free intracellular iron or iron-containing enzymes react with oxygen and lipids that contain polyunsaturated fatty acids (PUFAs). This results in high levels of membrane lipid peroxidation that induces cell death [3]. Yusuke H et al. [4] discovered that as lipid peroxides gradually accumulate on the cell membrane, the membrane tension rises, activating mechanically gated Piezo1 ion channels and transient receptor potential (TRP) ion channels. This leads to an influx of Na^+^ and Ca^2+^ accompanied by osmotic cell swelling. This ultimately results in cell membrane rupture. Ferroptosis was first reported in 2012, and accumulating evidence indicates that ferroptosis is extensively implicated in the pathophysiological process of IS and is one of the key events during brain ischemic injury. The levels of iron ions and the extent of lipid peroxidation in infarcted brain regions are significantly elevated in comparison with those in normal brain tissue. Numerous ferroptosis inhibitors can alleviate brain cell damage caused by ischemia and hypoxia to varying degrees and enhance neurological function [5]. TCM has significant curative effects on IS, and it has been shown to effectively improve the brain cell survival rate after IS and enhance neurological function, and it is characterized by multiple targets, multiple pathways, and high safety [6,7]. Studies have shown that ferroptosis is a target of TCM for IS treatment. TCM can regulate ferroptosis by alleviating iron overload [8], reducing reactive oxygen species production [9], and modulating lipid synthesis [10], thereby reducing brain tissue damage.

Network pharmacology is an emerging discipline based on systems biology theory that uses network analysis of biological systems, multi-target drug molecule design, and specific signal node selection. Machine learning is a branch of artificial intelligence that involves building mathematical models to learn patterns and relationships within data to predict new data or assist in decision-making. It has a wide range of potential applications for the study of complex disease mechanisms and the prediction of new therapeutic targets [11]. However, it is necessary to carry out a more systematic and profound exploration of ferroptosis regulation for IS treatment by numerous TCMs and their active constituents at the mechanistic level. This investigation is grounded in TCM, disease databases, and network analysis platforms, and we examine the correlations among the intersection targets of IS and ferroptosis, related small molecules, and TCM. We then use molecular docking technology to validate the binding stabilities of small molecules and target proteins, thereby offering theoretical support for a further all-round and systematic exploration of the molecular mechanism of TCM for ferroptosis regulation and broadening the clinical application domain of TCM.

In summary, we ultimately identified eight core targets of TCM interventions for ferroptosis in IS from which we screened out 413 small molecules and 301 TCMs. The properties, flavors, channels, and functions indicated that the TCMs were primarily cold, warm, bitter, and pungent in taste and that they primarily enter the liver meridian, with the primary functions being heat-clearing and deficiency-tonifying. We also confirmed the core roles of “*Salvia miltiorrhiza*, *Uncaria rhynchophylla*, and *Ginkgo biloba*” in IS treatment, providing a reference for research related to ferroptosis in IS.

## Materials and methods

**Fig 1:**
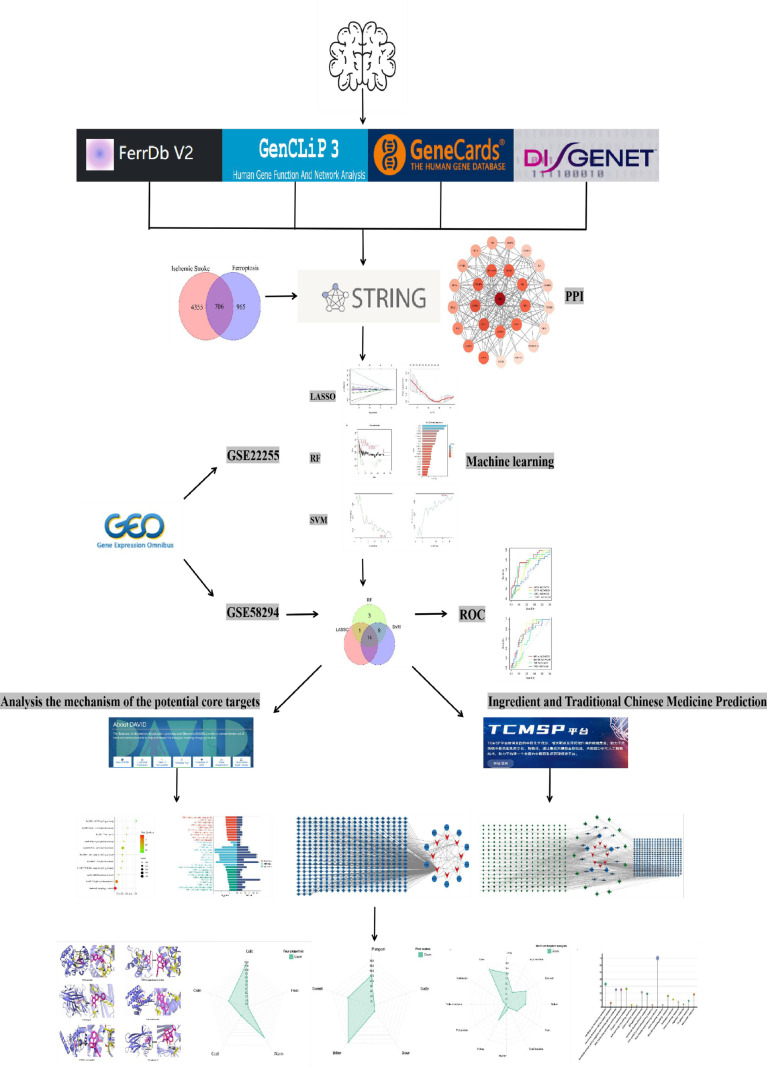
Research flowchart.

This study was based on information from databases such as GeneCards, GeneCLiP3, FerrDdb, DisGeNET, and GEO to obtain the ferroptosis targets related to IS. A protein-protein interaction (PPI) analysis was conducted, and machine learning screening validation was performed on potential targets. The DAVID database was then used to analyze the mechanisms of potential targets, and the TCMSP database was used to obtain related compounds and the TCMs. Molecular docking technology was used to verify the binding activities of compounds and core components of TCMs with the identified targets. The properties, flavors, meridian tropism, and therapeutic effects of the candidate TCMs were then analyzed and statistically evaluated. The detailed process is shown in **Fig 1**. The databases and software involved in this study and their corresponding websites can be found in **Table 1**.

**Table 1:** Database and analysis tools.

Database/Software	Website
Ferrdb V2 [12]	http://www.zhounan.org/ferrdb
GeneCards [13]	http://www.genecards.org
GeneCLiP3 [14]	http://ci.smu.edu.cn/genclip3/
DisGeNet [15]	http://www.disgenet.org/
BioLadder	https://www.bioladder.cn/
STRING [16]	https://string-db.org/
DAVID [17]	https://david.ncifcrf.gov/
Uniprot [18]	https://www.uniprot.org/
TCMSP [19]	https://www.tcmsp-e.com/#/home
GEO [20]	https://www.ncbi.nlm.nih.gov/geo/
PDB	https://www.rcsb.org/
Chiplot	https://www.chiplot.online/
SRplot [21]	https://www.bioinformatics.com.cn/
R 4.2.0	
Autodock vina 1.2.1	
PyMOL 1.8.5	
Cytoscape 3.10.2	
AutoDockTools 1.5.6	

### Acquisition of targets highly correlated with ferroptosis

The keyword, “Ferroptosis,” was searched for in the FerrDb, GeneCards, and GeneCLiP3 databases. The targets obtained from the three databases were combined, and the duplicates were removed to obtain the strongly related ferroptosis targets.

### Acquisition of IS disease targets

The GeneCards and DisGeNet databases were used to search for the keyword “Ischemic Stroke.” The filtering criterion for the Genecard database was set to a score value ≥ 75%. The targets obtained from the two databases were combined, and then the duplicates among them were removed to obtain the disease targets of IS.

### The potential targets of ferroptosis in IS

The above-mentioned targets related to ferroptosis and the IS targets were imported into BioLadder to construct a Venny diagram to obtain the potential targets of ferroptosis in IS. The STRING database was used to conduct a string analysis on the potential targets. In the multiple proteins module, the species (Organism) was set to “Homo sapiens,” and the minimum interaction score threshold was 0.9. The results were downloaded in tsv format, and the protein interaction network results were imported into Cytoscape 3.10.2 software for visualization. The network topology properties were analyzed using the Network Analyzer plugin, and a PPI network was drawn using a threshold five times the median degree value.

### Machine learning and validation

The transcriptome sequencing data related to IS were retrieved from the GEO database, and after filtering, the GSE22255 dataset [22] on the GPL570 platform and the GSE58294 dataset [23] were selected as the training set and validation set for machine learning, respectively. The GSE22255 dataset included data obtained from 20 healthy controls and 20 IS patient tissue samples. The GSE58294 dataset included data obtained from 23 healthy controls and 69 IS patient tissue samples, with the selected datasets having complete gene expression profiles and no ethical or moral issues or other conflicts of interest. The “RandomForest” R package was used for the Random Forest (RF) analysis, mtry was set to default values, and the threshold for ntree was set to 500. The “Glmnet” R package was used to apply the L1 regularization, and a minimum lambda of 0.01494666 was used as the threshold for the least absolute shrinkage and selection operator (LASSO) analysis. The “Caret” R package was used to select parameters for all classifiers, and a training dataset with five-fold cross-validation was used for the support vector machine (SVM) analysis. Finally, the overlapping genes from the LASSO, SVM, and RF algorithms were considered as potential core targets. Based on the GSE58294 dataset, a receiver operating characteristic (ROC) curve was constructed to validate the gene expression levels of potential core targets. An area under the curve (AUC) greater than 0.5 indicated that the results were reliable [24,25].

### Analysis of the mechanisms of the potential core targets

DAVID is a database that is focused on gene function annotation and data analysis. It provides a wealth of gene information that includes gene expression, functional classification, and pathway annotation. We used the DAVID database to implement a Gene Ontology (GO) functional enrichment analysis and a Kyoto Encyclopedia of Genes and Genomes (KEGG) enrichment pathway analysis on the obtained potential targets. The obtained data were imported into the online platform of SRplot to produce a three-in-one diagram of the biological process (BP), cellular component (CC), molecular function (MF), and the KEGG bubble chart.

### Screening candidate compounds and construction of the target-compound network

The potential core targets were then imported into the Uniprot database to obtain the full names of their target proteins. The Traditional Chinese Medicine Systems Pharmacology Database and Analysis Platform (TCMSP) was used to screen compounds related to the targets with the following criteria: drug-likeness (DL) not less than 0.18 and oral bioavailability (OB) not less than 30%. The targets-compounds network was then constructed using Cytoscape 3.10.2.

### Screening of candidate TCM and construction of the targets-compounds TCM network

With the assistance of the TCMSP, candidate TCMs that corresponded to the compounds related to the targets were screened and imported into the Cytoscape 3.10.2 for the construction of the targets-compounds-TCM network. In accordance with the “Pharmacopoeia of the People’s Republic of China: Volume I” (2020 Edition) and the “Science of Chinese Materia Medica,” a statistical analysis of the properties, flavors, meridian tropism, and efficacy of the candidate TCMs was conducted using Excel. In addition, radar charts of the properties and flavors and meridian tropism, as well as bar charts of the efficacies, were drawn.

### Molecular docking verification

A thorough analysis of the constructed target-compound network and the target-compound-TCM network was conducted, and the 10 compounds that possessed the highest degree values within both networks were meticulously selected to conduct 10 individual molecular docking validations, each targeting eight central proteins. This was done to preliminarily examine the binding activities between the potential core targets of ferroptosis in IS and the candidate compounds and core components of the candidate TCMs.

The 3D structure of the key protein molecules from the protein database (PDB) was obtained, and PyMOL software was used to remove water molecules and ligands from each protein and facilitate conversion into a PDBQT file. The MOL2 files of small molecules with high degree values were downloaded from the TCMSP. The processed protein receptor and corresponding small molecule ligands were imported into AutoDockTools 1.5.6 software, and the necessary preprocessing, such as hydrogenation, was performed. AutoDock Vina was then used for molecular docking. PyMOL software was used to visualize the combinations with strong binding capabilities.

## Results

### Ferroptosis-related targets in IS and validation

**Fig 2:**
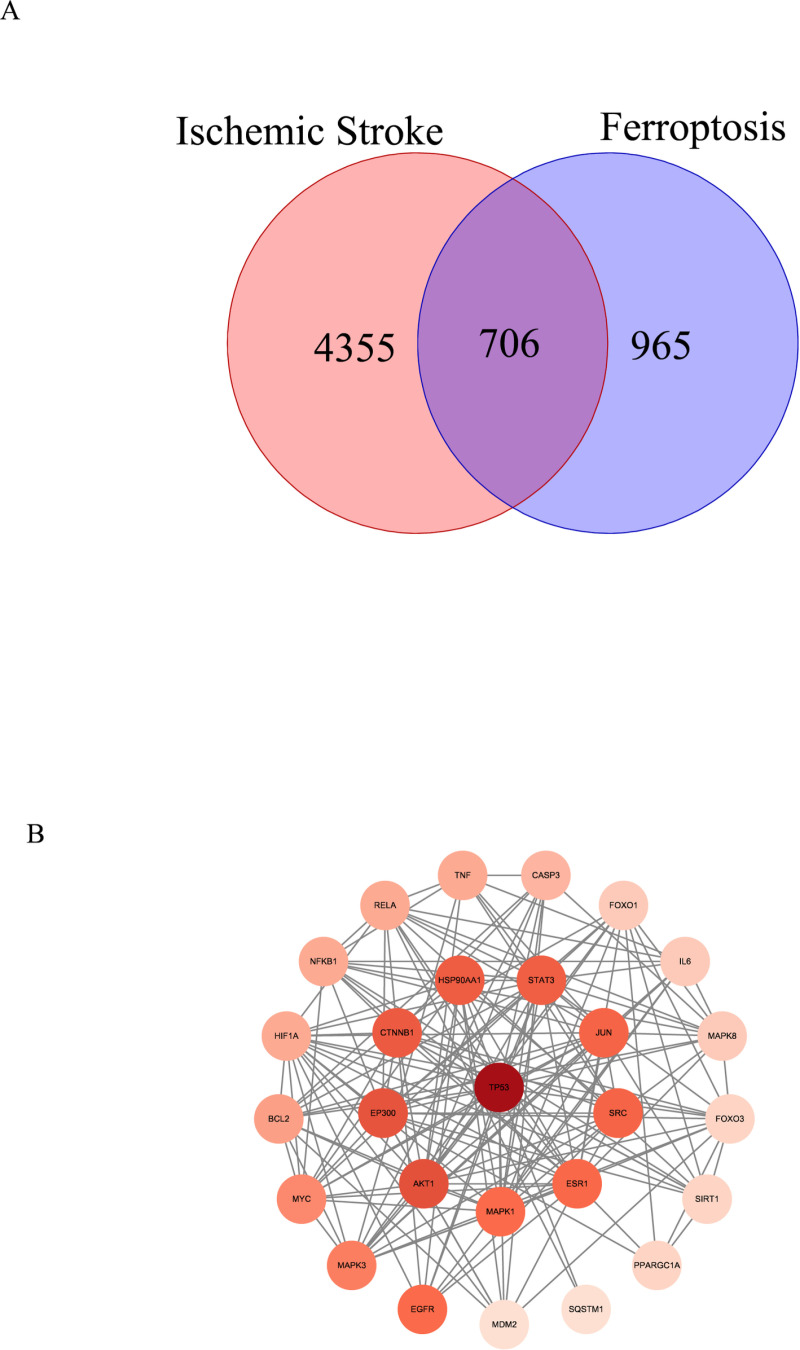
Venn diagram and protein–protein interaction (PPI) networks. **(A)** Venn diagram: red represents ischemic stroke, blue represents ferroptosis, and purple represents intersecting targets. **(B)** PPI networks of 27 potential targets associated with ischemic stroke in ferroptosis. The intensity of the color corresponds to the degree value, with darker colors indicating higher values.

**Fig 3:**
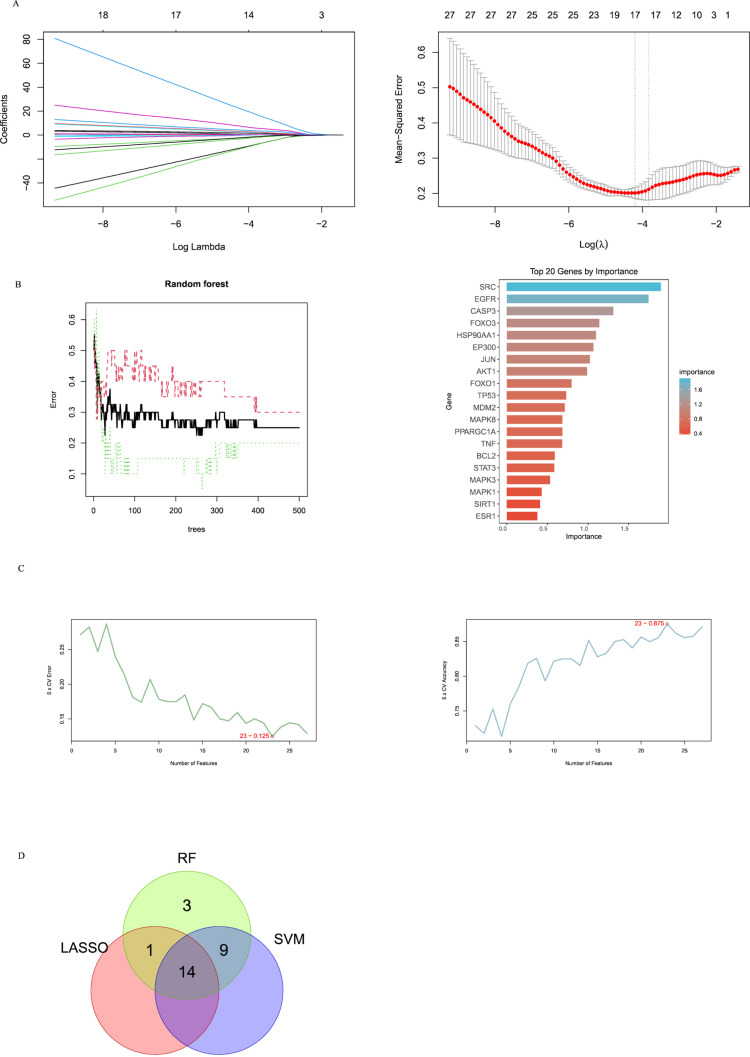
Screening hub genes by machine learning. **(A)** LASSO regression algorithm. **(B)** RF algorithm. **(C)** SVM algorithm. **(D)** Venn diagrams for the three algorithms. LASSO, Least Absolute Shrinkage and Selection Operator; SVM, Support Vector Machine; RF, Random Forest.

Through database retrieval, 1671 targets related to ferroptosis and 5061 targets related to IS were obtained, with 706 overlapping targets between the two. The String analysis results were imported into Cytoscape 3.10.2 software, where targets with a degree value ≥25 (five times the median) totaled 27 (**Fig 2**). The LASSO regression analysis selected 15 predictive genes from statistically significant variables, RF identified 27 predictive genes, and the SVM algorithm recognized 23 predictive genes. There were 14 overlapping genes identified by these three algorithms: RAC-alphaserine/threonine-proteinkinase (AKT1), cateninbeta-1, epidermal growth factor receptor (EGFR), histone acetyltransferase p300, estrogen receptor (ESR1), forkhead box protein O1(FOXO1), forkhead box protein O3, hypoxia-inducible factor 1-alpha (HIF1A), mitogen-activated protein kinase 8 (MAPK8), peroxisome proliferator-activated receptor gamma coactivator 1-alpha, NAD-dependent protein deacetylase sirtuin-1, sequestosome-1, tumor necrosis factor (TNF), and cellular tumor antigen p53 (TP53) (**Fig 3**).

### Analysis of the potential core target mechanisms

**Fig 4:**
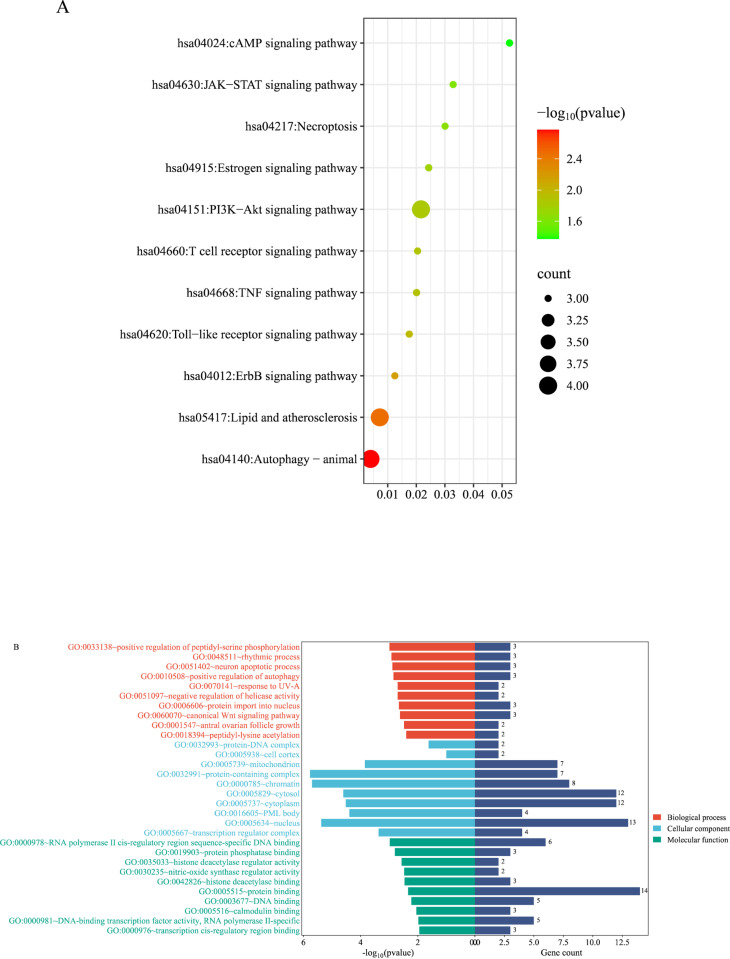
Kyoto Encyclopedia of Genes and Genomes and Gene Ontology enrichment analyses.

The potential core targets were imported into the DAVID database for the GO and KEGG analyses, and these were used to visualize the top-ranked targets from the GO and KEGG analyses (**Fig 4**). The GO enrichment analysis results for the core targets indicated that the BPs were primarily associated with neuronal apoptosis, positive autophagy regulation, positive peptide-serine phosphorylation regulation, and positive peptide-lysine acetylation regulation. The CCs primarily involved protein-DNA complexes, cytosol, and cytoplasm. The MFs were primarily related to protein phosphatase binding, histone deacetylase binding, and sequence-specific DNA binding of RNA polymerase II. The KEGG enrichment analysis results showed that the primary pathways involved included lipid and atherosclerosis signaling pathways, the ErbB signaling pathway, the Toll-like receptor signaling pathway, the TNF signaling pathway, and the PI3K-Akt signaling pathway. The functional annotations for the statistically significant enriched entries are shown in **Table 2**.

**Table 2:** Results of the Kyoto Encyclopedia of Genes and Genomes enrichment analysis of 14 core targets.

ID	Description	P value	Count	GeneID
hsa04140	Autophagy - animal	0.001704872388532211	4	MAPK8, AKT1, HIF1A, SQSTM1
hsa05417	Lipid and atherosclerosis	0.0034328091324413454	4	MAPK8, AKT1, TNF, TP53
hsa04012	ErbB signaling pathway	0.00680611828757366	3	MAPK8, AKT1, EGFR
hsa04620	Toll-like receptor signaling pathway	0.010753686080597916	3	MAPK8, AKT1, TNF
hsa04668	TNF signaling pathway	0.012721538563992694	3	MAPK8, AKT1, TNF
hsa04660	T cell receptor signaling pathway	0.013340653715608188	3	MAPK8, AKT1, TNF
hsa04151	PI3K-Akt signaling pathway	0.014340052599463091	4	AKT1, FOXO3, TP53, EGFR
hsa04915	Estrogen signaling pathway	0.017092844079808697	3	AKT1, ESR1, EGFR
hsa04217	Necroptosis	0.022018516577547314	3	MAPK8, TNF, SQSTM1
hsa04630	JAK-STAT signaling pathway	0.024407699486533888	3	EP300, AKT1, EGFR
hsa04024	cAMP signaling pathway	0.04216750230581914	3	MAPK8, EP300, AKT1

### Construction of a target-compound interaction network

**Fig 5:**
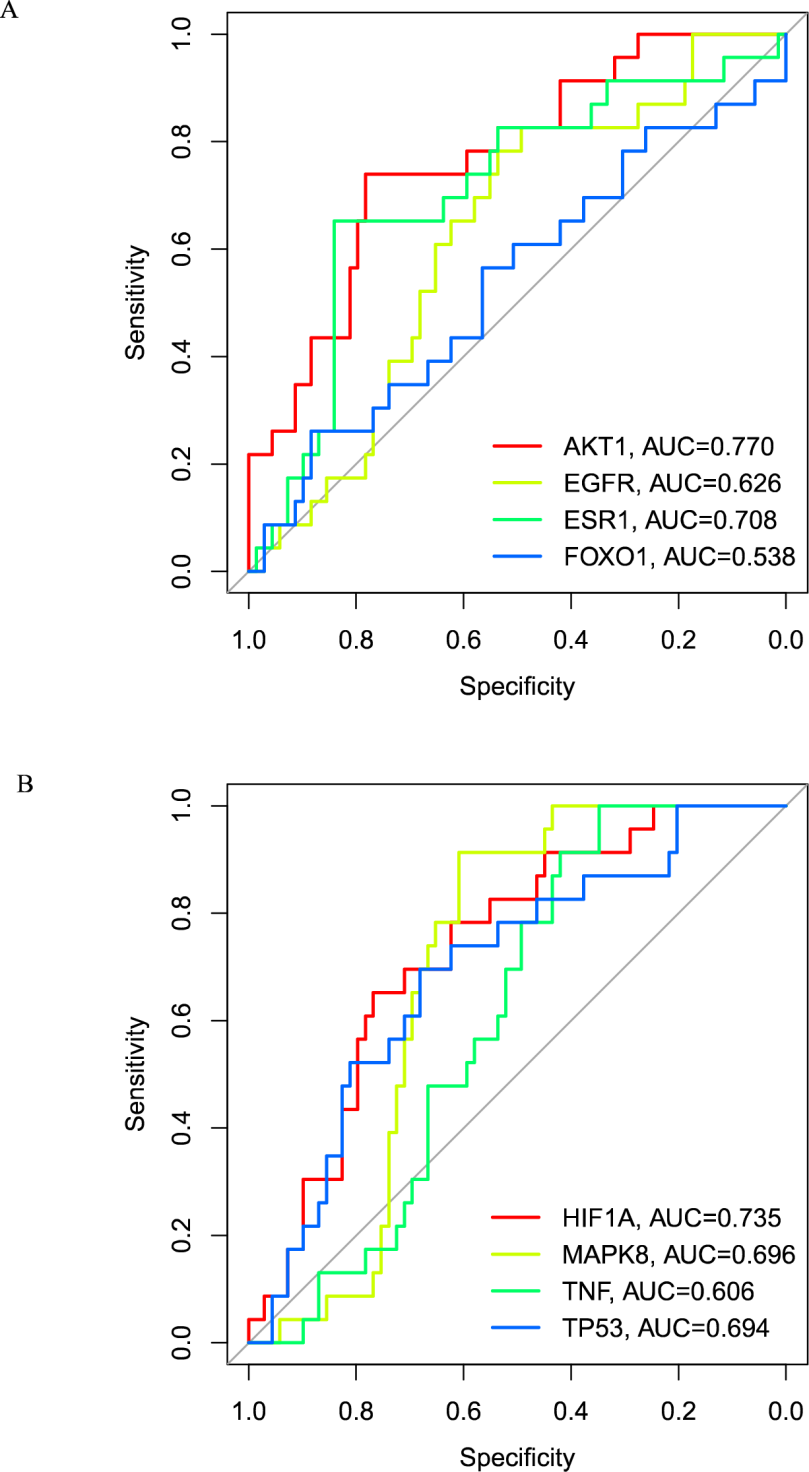
Receiver operating characteristic curve analysis. Hub genes in the GSE58294 dataset were analyzed using the receiver operating characteristic curves.

**Fig 6:**
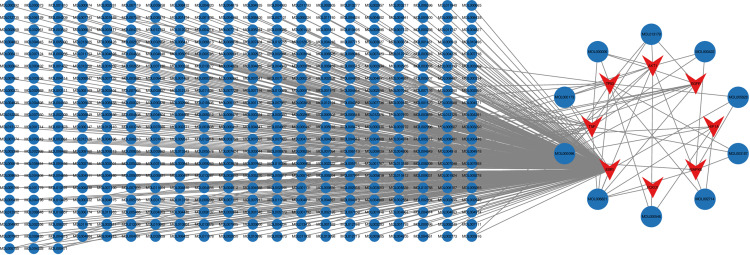
Visualization network of potential target and compound matching. Red nodes represent targets, and blue nodes represent compounds. The top 10 compounds are highlighted on the right side of the network diagram.

Fourteen potential targets of ferroptosis in IS were screened in the TCMSP using the criteria of DL ≥ 0.18 and OB ≥ 30%. Eight targets were obtained and considered as the central targets for the treatment of IS ferroptosis by TCM. The GSE58294 dataset was used to construct the ROC curves to evaluate the accuracy of these eight targets. The results showed that the AUC values for AKT1, EGFR, ESR1, FOXO1, HIF-1A, MAPK8, TNF, and TP53 were 0.77, 0.626, 0.708, 0.538, 0.735, 0.696, 0.606, and 0.694, respectively. These results indicated high accuracy and proved that these eight targets are central targets for the treatment of IS ferroptosis using TCM interventions (**Fig 5**). The above central targets corresponded to 413 compounds that were defined as candidate compounds for IS ferroptosis. The core targets and candidate compounds were imported into Cytoscape 3.10.2 software to construct a target-compound network (**Fig 6**). There are a total of 421 nodes and 447 edges in the figure, and the top 10 compounds ranked by the degree value are shown in **Table 3**.

**Table 3:** Top 10 compounds ranked by degree value in the target-compound interaction network.

Name	MOL	Degree
(-)-Epigallocatechin-3-gallate	MOL006821	8
Quercetin	MOL000098	5
Fisetin	MOL013179	4
Wogonin	MOL000173	4
Luteolin	MOL000006	4
Nobiletin	MOL005828	3
Triptolide	MOL003187	3
Baicalein	MOL002714	3
Diosgenin	MOL000546	3
Kaempferol	MOL000422	3

### Construction of a targets-compounds-TCM network

**Fig 7:**
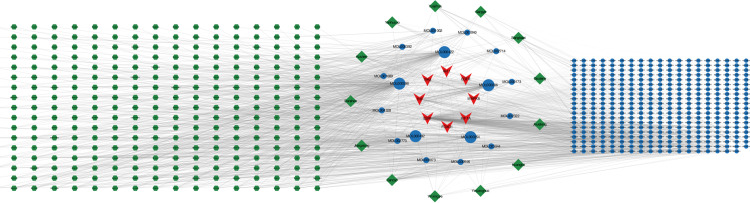
Visualization of the target-compound-TCM network. The red nodes represent the target, the blue nodes represent the compound, and the green nodes denote the TCM. At the center of the network diagram, the TCMs and small molecules with the highest correlations are highlighted.

A query of the TCMSP found a total of 301 TCMs that corresponded to 413 compounds. In accordance with the Pharmacopoeia of the People’s Republic of China: Part I (2020 edition) and Chinese Medicine, standardization was applied to the nomenclature, properties, tastes, and meridian tropism of the TCMs, encompassing a total of 301 TCMs. Subsequently, a targets-compounds-TCM network was constructed using Cytoscape 3.10.2 (**Fig 7**). The top 10 small molecules and TCMs ranked by the degree values are shown in **Tables 4 and 5**.

**Table 4:** Top 10 compounds ranked by degree value in the targets-compounds-TCM network.

Name	MOL	Degree
Quercetin	MOL000098	194
Kaempferol	MOL000422	137
Luteolin	MOL000006	97
(+)-Catechin	MOL000492	42
Isorhamnetin	MOL000354	41
Beta-carotene	MOL002773	30
Ent-Epicatechin	MOL000073	28
Naringenin	MOL004328	24
Acacetin	MOL001689	22
Formononetin	MOL000392	19

**Table 5:** Top 10 Chinese medicines ranked by degree value.

Name	Degree	Four properties	Five tastes	Meridian tropism	Efficacy analysis
Gancao	79	Calm	Sweet	Heart, Lung, Spleen, Stomach	Tonifying herbs
Jiangxiang	24	Warm	Pungent	Liver, Spleen	Hemostatic herbs
Danshen	22	Cold	Bitter	Heart, Liver	Blood-moving herbs
Kushen	15	Cold	Bitter	Heart, Liver, Stomach, Large intestine, Bladder	Heat-clearing and detoxifying herbs
Yanhusuo	14	Warm	Pungent, Bitter	Liver, Spleen	Blood-moving herbs
Yuejihua	12	Warm	Sweet	Liver	Blood-moving herbs
Sangye	12	Cold	Bitter, Sweet	Lung, Liver	Exterior-releasing herbs
Sangbaipi	12	Cold	Sweet	Lung	Phlegm-resolving and antitussive Herbs
Yinyanghuo	10	Warm	Pungent, Sweet	Liver, Kidney	Tonifying herbs
Yinxingye	10	Calm	Sweet, Bitter	Heart, Lung	Blood-moving herbs
Jixueteng	10	Warm	Bitter, Sweet	Liver, Kidney	Blood-moving herbs
Huangbo	10	Cold	Bitter	Kidney, Bladder, Large intestine	Heat-clearing and detoxifying herbs
Gouteng	10	Cool	Sweet	Liver, Pericardium	Liver-calming and wind-extinguishing herbs

### Properties, tastes, meridian tropism, and efficacy analysis of the candidate TCMs

**Fig 8:**
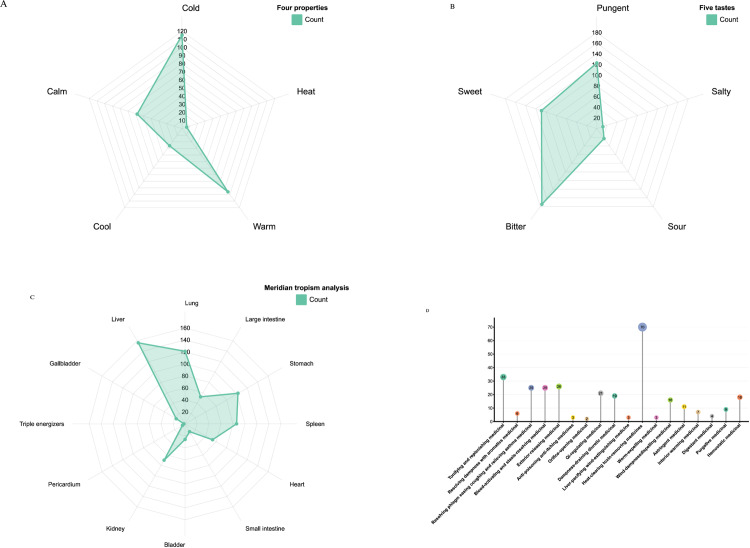
Properties, tastes, meridian tropism, and efficacy analysis of the candidate TCMs.

Excel was used to conduct frequency statistical analysis of the four properties, five tastes, meridian tropism, and effects of the candidate TCMs. This revealed that the candidate herbs were primarily cold-natured [115 instances (frequency of 38%)], warm-natured [96 instances (frequency of 32%)], bitter taste [175 instances (frequency of 40%)], pungent taste [123 instances (frequency of 28%)], enter the liver meridian [156 instances (frequency of 22%)], heat-cleaning medicinal [70 instances (frequency of 23%)], and tonify deficiency [33 instances (frequency of 11%)] (**Fig 8**).

### Molecular docking

The top 10 small molecules with the highest degree values were selected for the molecular docking validation based on the target-compound network and the target-compound-TCM network analysis results. In addition, we also selected three TCMs (*Salvia miltiorrhiza*, *Ginkgo biloba* leaves, *Uncaria rhynchophylla*) with high degree values in the network that have clinical applications related to stroke. The core components of these TCMs were docked with central targets to further verify that ferroptosis is indeed one of the targets for TCM treatment of IS. The selected molecules and target information are shown in **Tables 6 and 7**.

**Table 6:** Docking information of the small molecules.

MOL ID	Name	MOL ID	Name
MOL000098	Quercetin	MOL006821	(-)-epigallocatechin-3-gallate
MOL000422	Kaempferol	MOL013179	Fisetin
MOL000006	Luteolin	MOL000173	Wogonin
MOL000492	(+)-Catechin	MOL005828	Nobiletin
MOL000354	Isorhamnetin	MOL003187	Triptolide
MOL002773	Beta-carotene	MOL002714	Baicalein
MOL000073	Ent-Epicatechin	MOL000546	Diosgenin
MOL004328	Naringenin	MOL007154	Tanshinone II A
MOL001689	Acacetin	MOL011061	Ginkgolide B
MOL000392	Formononetin	MOL008486	Isorhynchophylline

**Table 7:** Target docking information.

Gene	PDB ID	Gene	PDB ID
AKT1	5AAR [26]	FOXO1	3CO6 [27]
EGFR	4Z21 [28]	ESR1	7BAA [29]
HIF1A	4H6J [30]	TNF	6OP0 [31]
MAPK8	4QTD [32]	TP53	7LIN [33]

**Fig 9:**
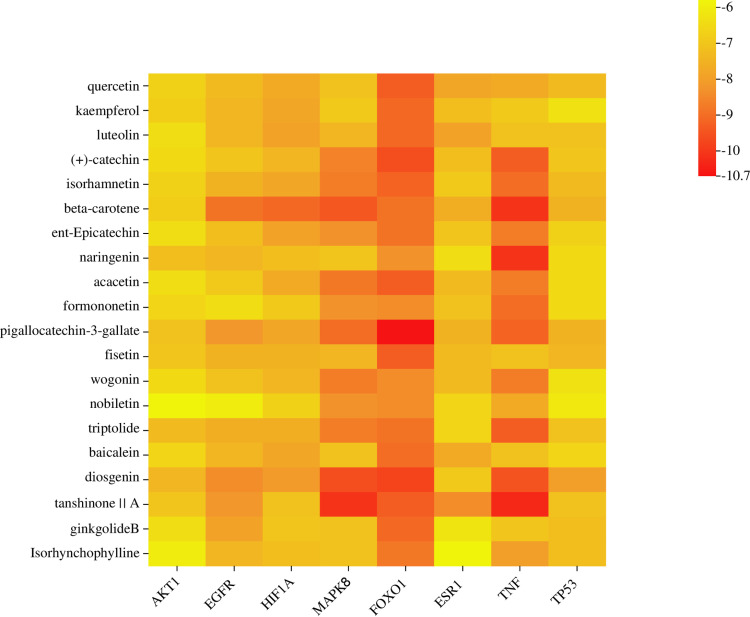
Molecular docking heat map of the core target interactions with the compounds.

**Fig 10:**
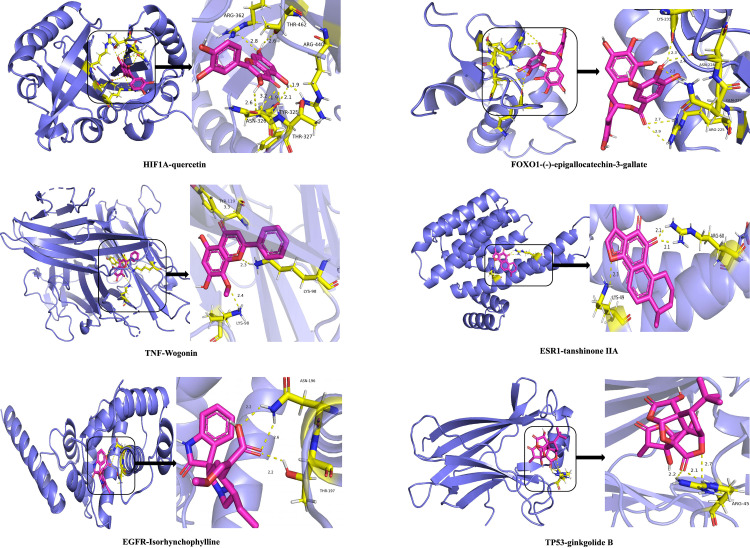
Docking results of the primary target related to ferroptosis in IS and its interaction with TCM compounds.

The results showed that all of the compounds had binding energies with the targets of less than −5.0 kcal/mol. This indicated strong binding activity between the compounds and the target. Beta-carotene had the lowest binding energy with five central targets, TNF, MAPK8, HIF-1A, EGFR, and FOXO1, with values of −10.1, −9.4, −9.1, −8.9, and −8.9 kcal/mol, respectively.

(-)-Epigallocatechin-3-gallate had the best binding with the FOXO1 target, with a binding energy of −10.7 kcal/mol. Tanshinone II A had a binding energy of −10.3 kcal/mol with the TNF target. The detailed molecular docking results are shown in **Fig 9**. Some molecular docking results are presented in **Fig 10**.

## Discussion

Ischemic stroke accounts for over 80% of all stroke subtypes and is a leading cause of long-term severe disability and mortality. Given the adverse side effects and significant limitations of thrombolytic therapy, the therapeutic outcomes for IS are generally poor, with a grim prognosis, making it imperative to further investigate the mechanisms of IS injury and explore novel treatment strategies. The essence of ferroptosis involves lipid peroxidation. The brain is abundant in PUFAs and has a low concentration of antioxidant enzymes; hence, it is particularly susceptible to free radical-induced neuronal damage. Consequently, the nervous system is intimately linked to ferroptosis. Although current studies have proven that the ferroptosis regulation by TCM is an important method to treat IS, its effective components, related TCMs, and molecular mechanisms remain unclear. This largely limits TCM use. Therefore, clarification of the TCM mechanism of action and finding new therapeutic targets are of great significance for TCM development and applications.

LASSO is a data mining method that filters out significant variables and constructs the best classification model by applying an L1 penalty to set the coefficients of less important variables to zero. It can effectively select the most important genes in a gene expression analysis for functional prediction or disease classification [34]. RF analysis is a machine learning method based on decision trees. By training RF models, the importance of each gene can be assessed, and the most relevant subset of genes related to the predictive target can be selected [35]. SVM is a powerful model for regression and classification that uses kernel functions to make an inseparable dataset separable, thereby achieving relatively ideal results for datasets that are not too complex and are of medium to small size [36]. However, even though these are traditional machine prediction models, the three aforementioned methods also have certain limitations [11]. For instance, when a model has a large number of features, LASSO is more susceptible to noise in the data. This leads to “overfitting” and poor performance on new, unseen data. RF has the characteristics of high accuracy and strong resistance to overfitting, but it often has high computational complexity that makes the prediction results difficult to interpret. SVM is not good at handling large-scale data. Given these limitations, many studies have chosen to apply three machine learning models, LASSO, RF, and SVM, simultaneously to screen and take the average of the results obtained [24,25,37]. This approach not only enhances the robustness of the gene selection and the generalization ability of model prediction but also facilitates the interpretation and understanding of the model and its potential application value [11].

We used PPI networks and machine learning in this study to identify eight key targets of TCM in treating IS via ferroptosis (AKT1, EGFR, ESR1, FOXO1, HIF-1A, MAPK8, TNF, and TP53). These targets corresponded to 413 TCM compounds and 301 traditional Chinese herbs. The statistical analysis indicated that TCM was predominantly used for its heat-clearing properties, primarily affecting the liver channel, with the prevalence of cold, warm, bitter, and pungent flavors. The results of this study provide a theoretical foundation to systematically investigate the molecular mechanisms by which TCM modulates ferroptosis, thereby expanding TCM clinical applications.

### Mechanistic analysis of ferroptosis targets in IS

The results revealed 14 potential core targets common to both IS and ferroptosis based on the analysis of potential targets associated with ferroptosis in IS. Among these, eight central targets were matched with significant Chinese medicine components in the TCMSP. Previous studies have demonstrated that eight targets, namely AKT1, EGFR, ESR1, FOXO1, HIF-1A, MAPK8, TNF, and TP53, engage in the regulation of ferroptosis by modulating inflammatory responses and intervening in lipid and iron ion metabolism and other mechanisms.

Excessive accumulation of iron ions within cells is one of the characteristics of ferroptosis. In fact, Fe^2+^ not only enhances lipid metabolism-related enzyme activities such as ACSL4 and lipoxygenase (LOX) but also directly participates in the lipid peroxidation process through the “Fenton reaction.” Studies have shown that both ESR1 and FOXO1 regulate iron ion metabolism and reduce excessive iron ion accumulation within cells. ESR1 promotes the ubiquitination and degradation of transferrin (TFR1) and increases SLC7A11 expression, thereby reducing the intracellular Fe^2+^ content and the level of membrane lipid peroxidation [38]. In contrast, FOXO1 activation inhibits NCOA4 expression, which facilitates the degradation of ferritin to release stored iron ions within cells, leading to ferroptosis [39].

Lipid metabolism is closely related to ferroptosis, and lipid peroxide accumulation is the core ferroptosis process. Studies have found that the activation of EGFR and MAPK8 can both increase the expression of Nrf2 protein in the lipid peroxidation reduction system [40]. Nrf2 is a key regulator of cellular oxidative stress responses that reduce the occurrence of neuronal ferroptosis by promoting the transcription of SLC7A11 and GPX4 genes during IS, thereby improving learning, memory, and cognitive functions in rats [41]. EGFR ubiquitination and degradation reduce Nrf2 expression and promote ferroptosis [42]. The TP53 gene encodes the tumor suppressor protein, P53, that plays a pivotal role in cellular responses to various stresses. Lu et al. [43] observed that increased P53 expression in the ischemic area in an animal model of brain ischemia led to decreased SLC7A11 levels. This resulted in a significant rise in ferroptosis in cells. Conversely, the inhibition of P53 expression might elevate SLC7A11 activity and mitigate ferroptosis, thereby ameliorating neurological symptoms. Additionally, P53 also heightens cellular susceptibility to ferroptosis by upregulating SAT1 gene expression, exacerbating cell damage [44]. AKT1 is a subtype of AKT. Researchers [45] have shown that it promotes the generation of monounsaturated fatty acids mediated by SREBP1/SCD1 and regulated by the AKT/mTORC1 pathway while reducing the PUFA content. However, it inhibits ACSL4 activity, blocks the conversion of PUFAs to PE, reduces lipid peroxide accumulation within cells, and ultimately inhibits ferroptosis [46].

**Fig 11:**
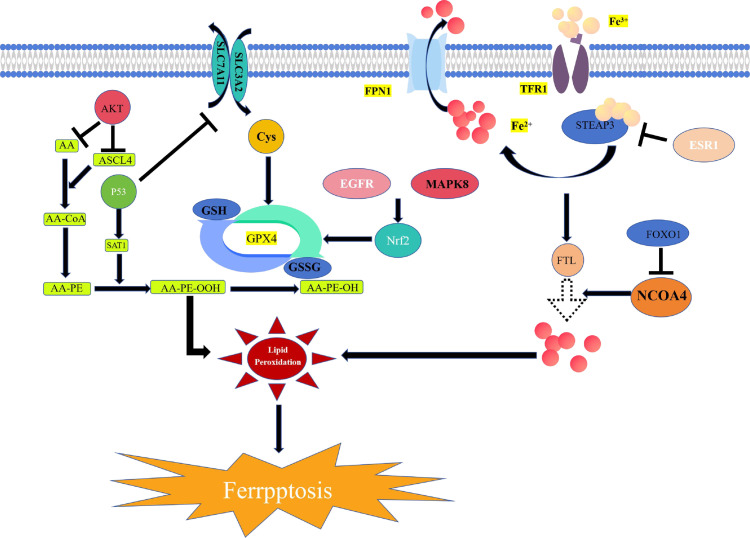
Ferroptosis mechanism illustration.

The hypoxic state and the inflammatory response induce brain cell ferroptosis after IS. Hypoxia-inducible factor HIF-1A is activated under hypoxic conditions and may inhibit ferroptosis occurrence by promoting mitochondrial autophagy, reducing mitochondrial redox homeostasis, limiting mitochondrial respiration, and restricting mitochondrial damage [47]. Zhou et al [48]. also found that under a hypoxic environment, HIF-1α promotes lactate generation, upregulates glutamate transporter SLC1A1 expression, and thus exerts an inhibitory effect on ferroptosis. TNF is a key pro-inflammatory cytokine in the mechanism of IS injury that is closely associated with ferroptosis. TNF-α induces ferroptosis by promoting the accumulation of reactive oxygen species (ROS), lipid peroxides, and iron ions, and once cells undergo ferroptosis, it further stimulates inflammation [49]. However, some studies have found that TNF-α may play a positive role in certain cells, such as macrophages and synovial fibroblasts, where it can significantly enhance the activity of SystemXc- and reduce the occurrence of ferroptosis [50,51] (**Fig 11**).

### Prediction of ferroptosis after stroke in TCM

This study identified 413 types of TCM components using reverse targeting and validation of the top 10 small molecules with the highest degree values in the target-compound network and the target-compound-TCM network via molecular docking. The results demonstrated that the docking effects were satisfactory. Epigallocatechin-gallate (EGCG), quercetin (QRC), and wogonin have been found to bind to various targets with optimal effects, and this has drawn our attention. Previous research has indicated that they can markedly improve neuroinflammation induced by IS and exert neuroprotective effects [52–54]. They intervene in ferroptosis by reducing oxidative stress and inhibiting lipid peroxidation. QRC can restrain ROS production and activate the nuclear translocation of Nrf2, thereby enhancing GPX4 and SLC7A11 activities and accelerating the clearance of lipid peroxides [9]. EGCG, conversely, can promote cellular autophagy and enhance GPX4 activity to inhibit the occurrence of ferroptosis [8,55]. Wogonin is an important flavonoid monomer found in the TCM *Scutellaria baicalensis* that can specifically upregulate GPX4 activity to alleviate ferroptosis and inhibit the activities of iNOS and ALOX15 to reduce ferroptosis and exert anti-ischemia-reperfusion injury effects [10].

Among the 301 TCMs collected by reverse screening of active ingredients, those with pungent, warm, bitter, and cold properties were predominant. These primarily target the liver channel. In terms of efficacy, they primarily serve to clear heat and tonify deficiencies. To further explore the core TCMs, we constructed a target-compound-TCM network graph, screened the components using degree values, and combined the results with literature retrieval methods. Ultimately, we identified *Salvia miltiorrhiza*, *Ginkgo biloba* leaves, and *Uncaria rhynchophylla* as the core TCM combination for the treatment of ferroptosis in IS. The degree value indicated that *Salvia miltiorrhiza*, *Ginkgo biloba* leaves, and *Uncaria rhynchophylla* were among the top ranked and widely used in clinical practice [56]. Known as Danshen in China and first recorded as a “top-grade” herb in Sheng Nong’s Herbal Classic (A.D.10–200), *Salvia miltiorrhiza* has been used for treating cardiovascular diseases and neurasthenic insomnia for thousands of years [57]. Modern research has proven that [58] the primary component of *Salvia miltiorrhiza*, tanshinone II A, can promote Nrf2 expression in cells, reduce the intracellular ROS content, and reduce active iron, thereby inhibiting the occurrence of neuronal ferroptosis and improving the IS prognosis. According to classical Chinese herbal books and Chinese Pharmacopoeia, relieving cough, reducing phlegm, clearing poison, and relieving diarrhea are the primary pharmacological effects of *Ginkgo biloba* leaves [59]. Research has found that the primary component of *Ginkgo biloba* leaves, Ginkgolide B, reduces the release of intracellular iron ions and promotes GPX4 activity, thereby reducing brain ischemic injury [60]. *Uncaria rhynchophylla* is a TCM used for many years in China, and it is widely used to treat diseases of the central nervous system [61]. The primary component of *Uncaria rhynchophylla*, isorhynchophylline, promotes Mir-122-5p expression to inhibit TP53 protein activity, thereby upregulating SLC7A11 activity and protecting nerve cells from ferroptosis [62].

TCM posits that stroke arises from internal organ dysfunction, brain channel obstruction, and poor blood circulation. This investigation revealed that TCM for IS was primarily composed of heat-clearing and body-supplementing herbs, reflecting the concept of fortifying the body, strengthening deficient areas, eliminating pathogens, and unblocking channels. In TCM, “Qi, blood, and body fluids” are the material foundation of human life activities. Once Qi, blood, and body fluids become pathological, they will affect the life activities of the human body [63]. Brain channel blockages impede the nourishment of brain orifices by Qi, blood, and bodily fluids. This results in a loss of spiritual essence and spiritual damage. Furthermore, local stagnation and accumulation of Qi and blood can generate phlegm and blood stasis, and the prolonged presence may transform into fire toxin with exacerbating effects. In TCM theory, “fire” corresponds to the modern inflammatory response and microcirculation disorders, while “toxin” denotes pathological product accumulations. A build-up of lipid peroxides following IS post-ferroptosis aligns with the “fire toxin” theory. Therefore, the combination of TCM predictions with a literature research indicated that for the treatment of IS with syndrome differentiation, appropriate bitter and cold substances can be added to clear heat and detoxify. In addition, warming and tonifying should also be considered. On this basis, TCMs such as *Salvia miltiorrhiza*, *Ginkgo biloba* leaves, and *Uncaria rhynchophylla* can be used to reduce the accumulation of ROS and iron ions, prevent iron death of brain cells, and promote functional recovery.

## Conclusion

In summary, this study, we began with the targets of IS and ferroptosis. We concluded that genes such as AKT1, EGFR, ESR1, FOXO1, HIF-1A, MAPK8, TNF, and TP53 might be key genes in the ferroptosis of IS, and they are expected to become the primary targets for treating IS. In addition, compounds such as EGCC, QRC, wogonin, tanshinone II A, isorhynchophylline, and ginkgolide B were found to be important for ferroptosis regulation in IS treatment and could provide a reference for the development of new drugs for IS treatment. *Salvia miltiorrhiza*, *Ginkgo biloba* leaves, and *Uncaria rhynchophylla* were found to be the TCMs for ferroptosis regulation in IS treatment, and they can provide a reference for clinical TCM IS treatment. However, this study still has some shortcomings. First, the data used for analysis were relatively limited. Second, the predicted TCM components have been largely studied in animal models, although we did verify them with molecular docking. However, more clinical trials are required to further confirm our results.

## Supporting information

S1 File Molecular docking 1. (ZIP)

S2 File Molecular docking 2. (ZIP)

S3 File Molecular docking 3. (ZIP)

S4 File Molecular docking 4. (ZIP)

S5 File Molecular docking 5. (ZIP)

S6 File Molecular docking 6. (ZIP)

S7 File Core gene screening and herbal medicine acquisition. (ZIP)
