# Supplementary figures and images for: Ferroptosis regulation by traditional chinese medicine for ischemic stroke intervention based on network pharmacology and data mining
Source: PLoS One. 2025 Apr 16;20(4):e0321751. doi: 10.1371/journal.pone.0321751 (PMC12002469; doi:10.1371/journal.pone.0321751)

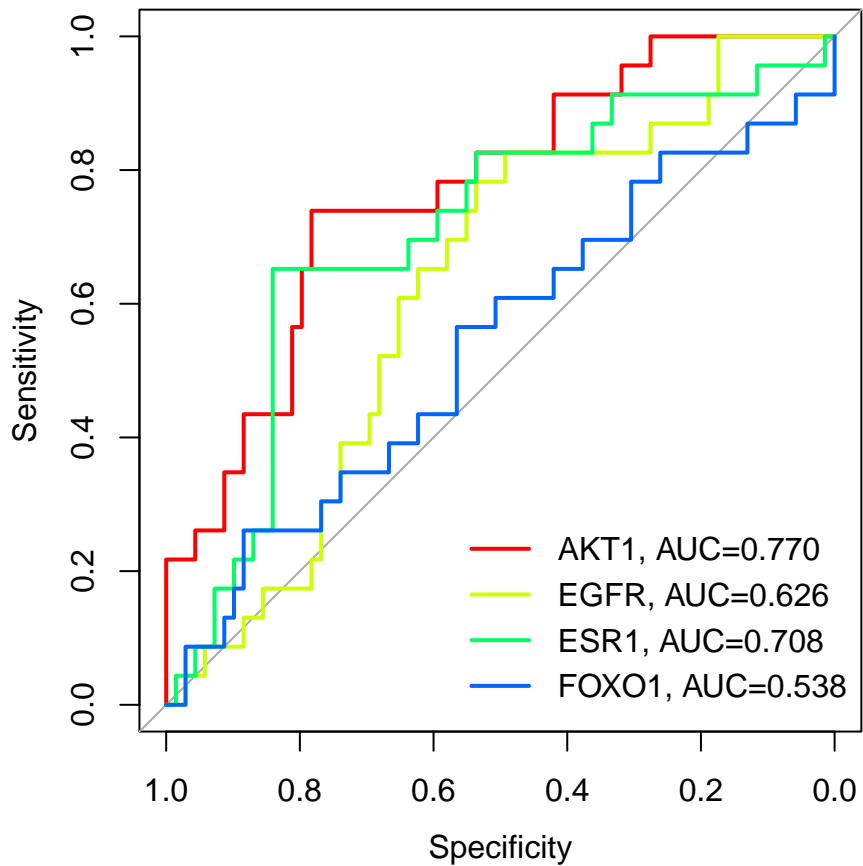

Supplement: S1 File — (ZIP) [file pone.0321751.s001.zip › 机器学习结果 27genes/GSE58294-1.pdf]

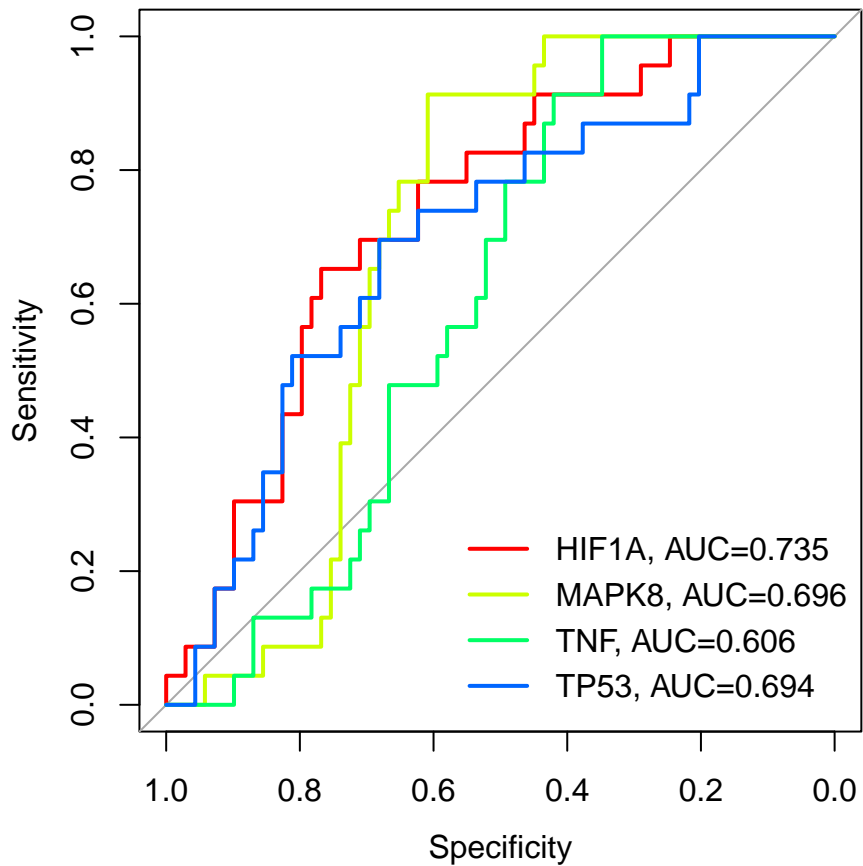

Supplement: S1 File — (ZIP) [file pone.0321751.s001.zip › 机器学习结果 27genes/GSE58294-2.pdf]

Coefficients

18

17

14

3

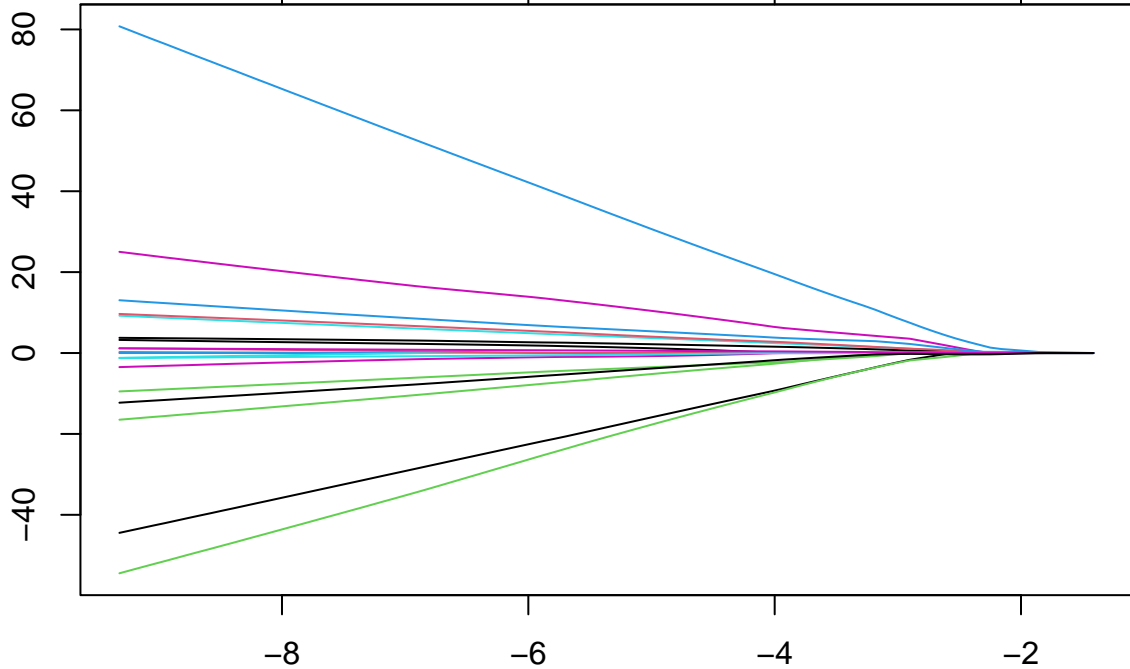

Log Lambda

Supplement: S1 File — (ZIP) [file pone.0321751.s001.zip › 机器学习结果 27genes/lasso-27genes/Rplot.pdf]

Mean-Squared Error

27 27 27 27 25 25 25 23 19 17 17 12 10 3 1

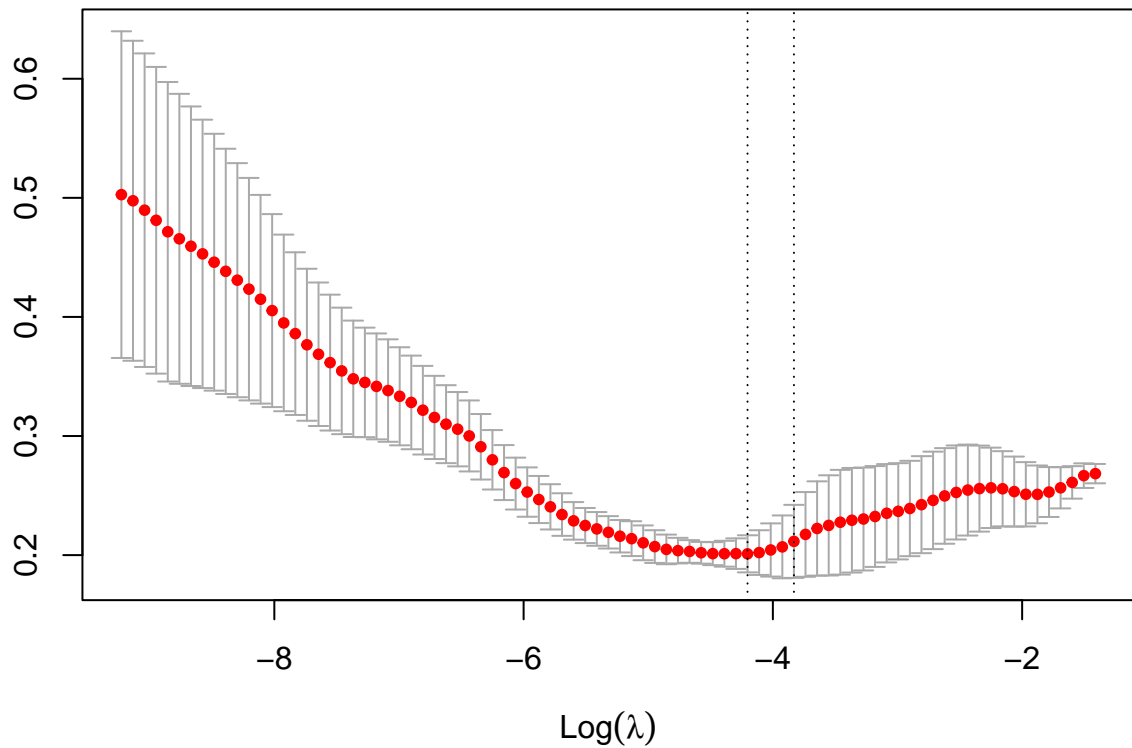

Supplement: S1 File — (ZIP) [file pone.0321751.s001.zip › 机器学习结果 27genes/lasso-27genes/Rplot01.pdf]

# Top 20 Genes by Importance

Gene

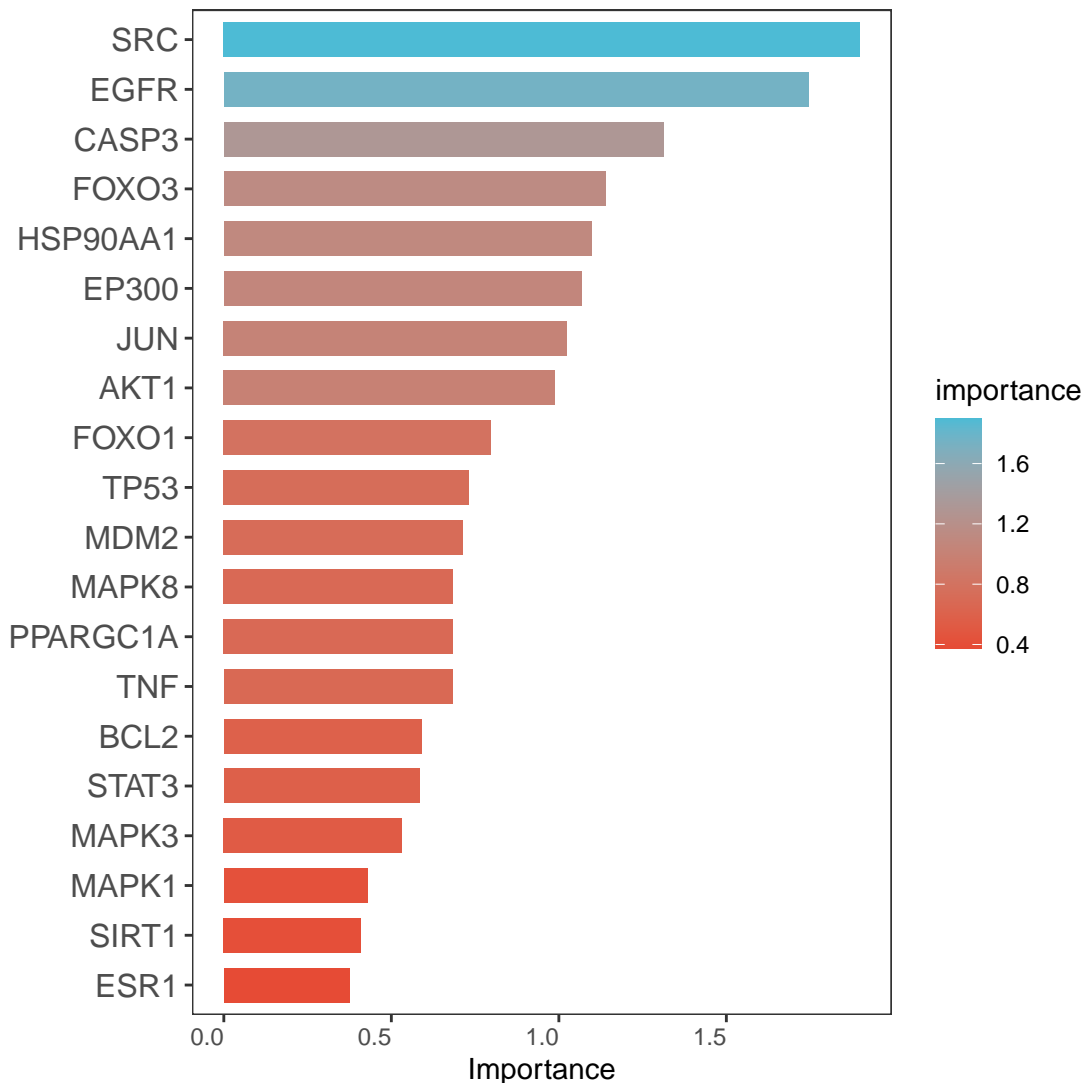

Supplement: S1 File — (ZIP) [file pone.0321751.s001.zip › 机器学习结果 27genes/RF-27genes/重要性基因20-1.pdf]

Gene

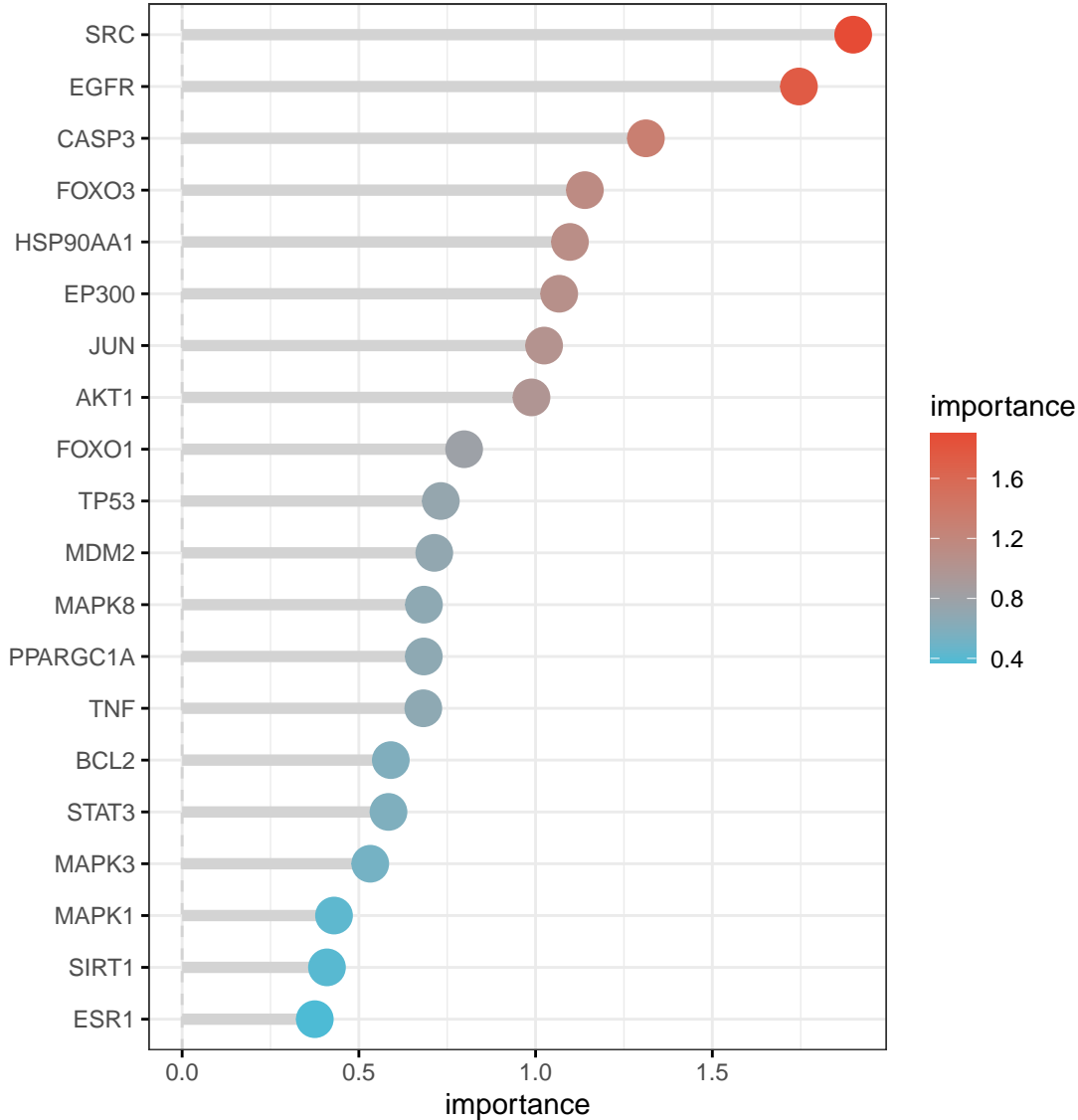

Supplement: S1 File — (ZIP) [file pone.0321751.s001.zip › 机器学习结果 27genes/RF-27genes/重要性基因20-2.pdf]

## Random forest

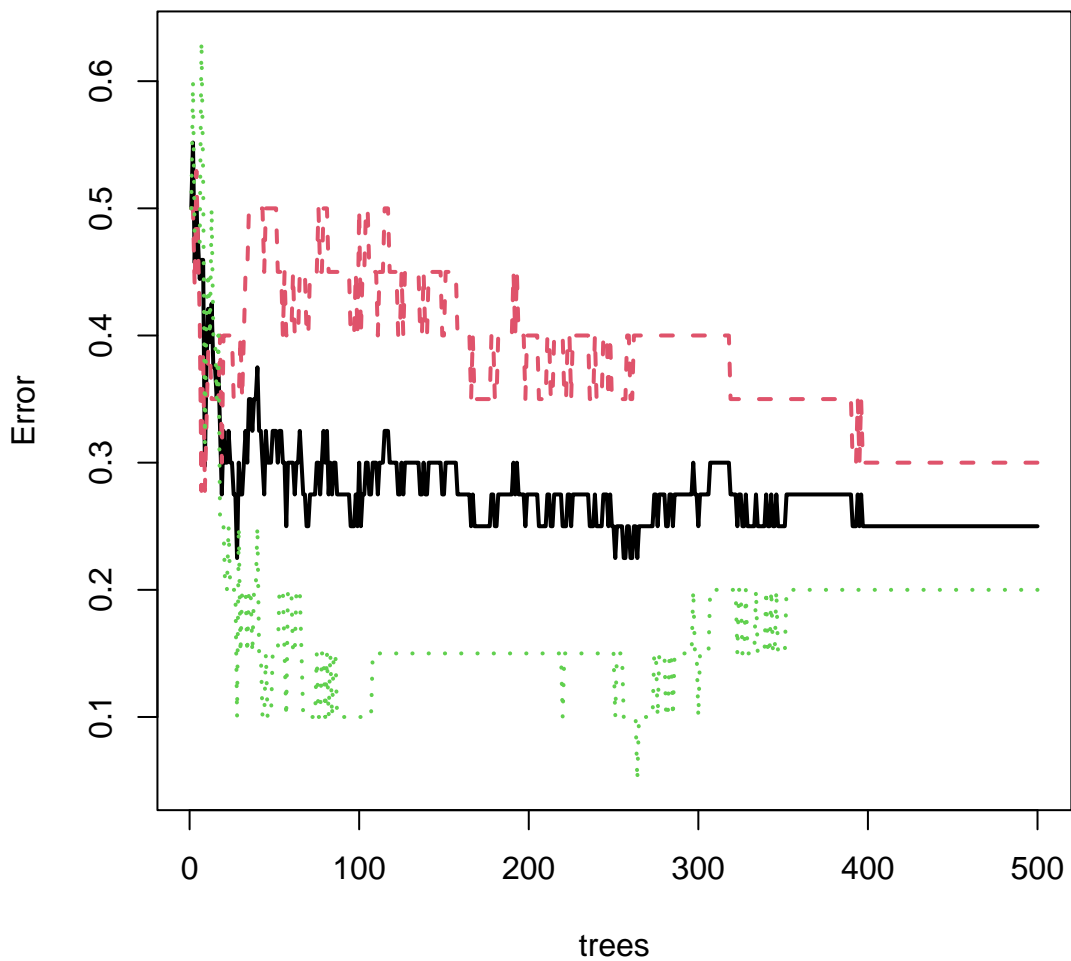

Supplement: S1 File — (ZIP) [file pone.0321751.s001.zip › 机器学习结果 27genes/RF-27genes/随机森林图.pdf]

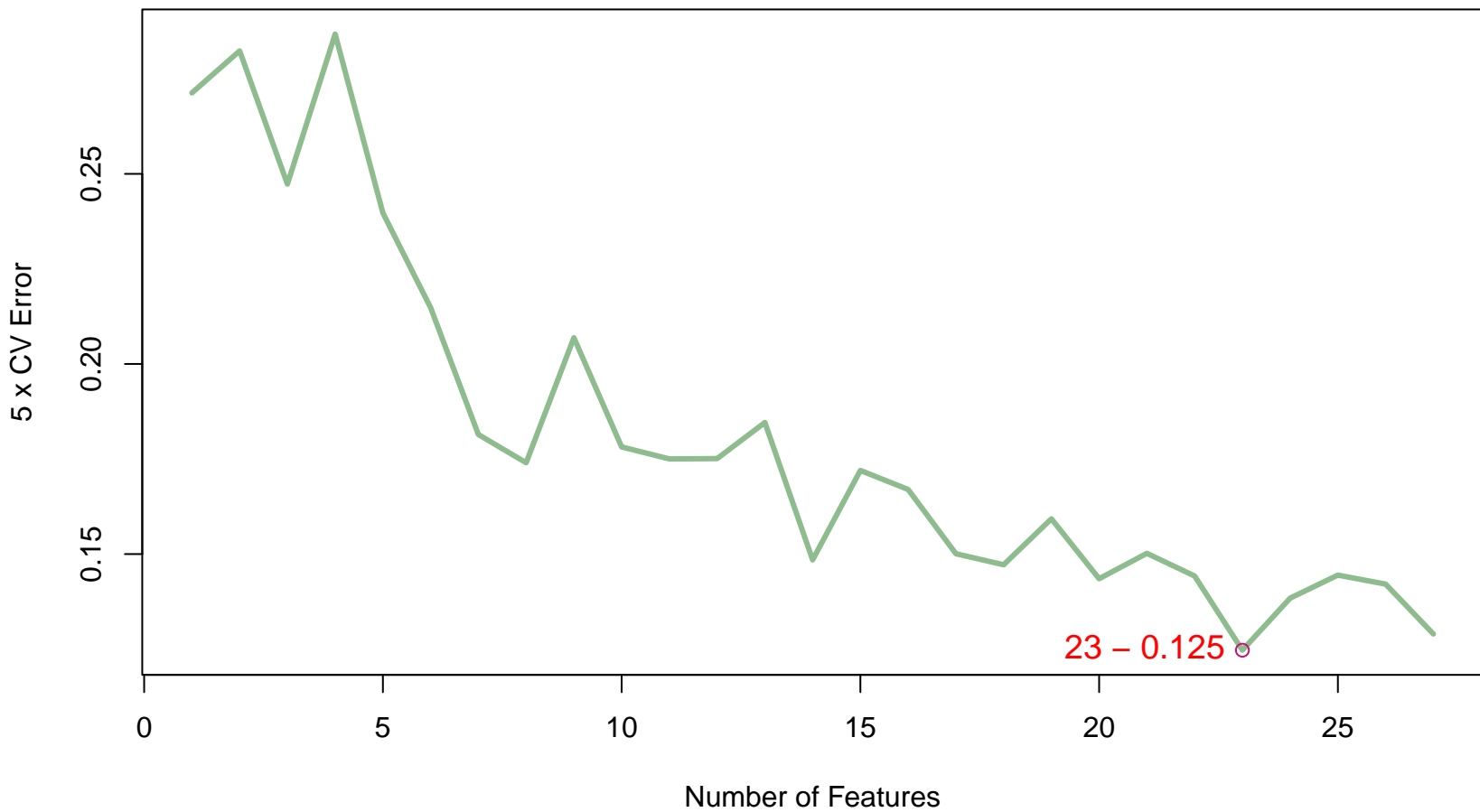

Supplement: S1 File — (ZIP) [file pone.0321751.s001.zip › 机器学习结果 27genes/SVM-27genes/Rplot.pdf]

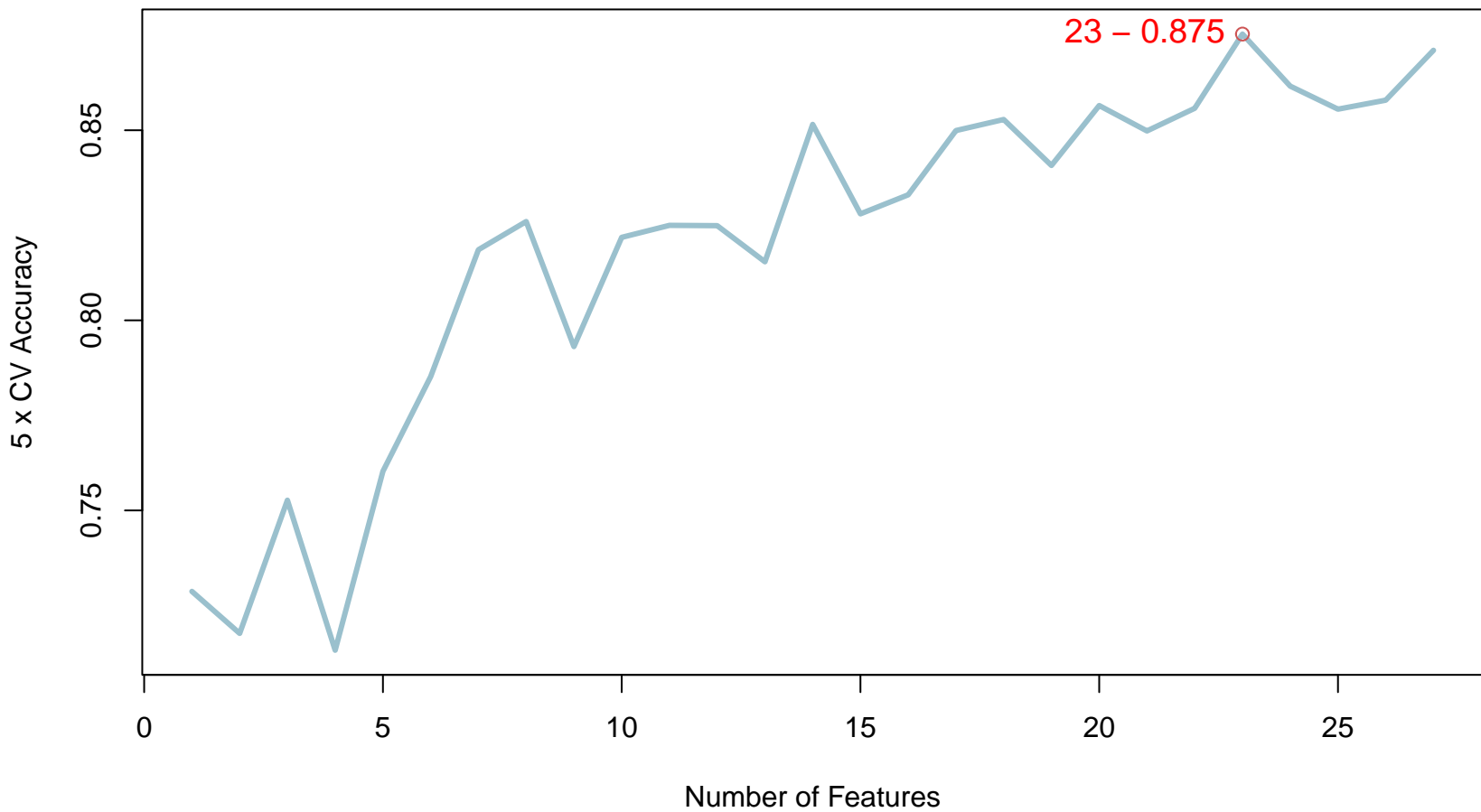

Supplement: S1 File — (ZIP) [file pone.0321751.s001.zip › 机器学习结果 27genes/SVM-27genes/Rplot01.pdf]

RF

3

LASSO

1

SVM

9

14

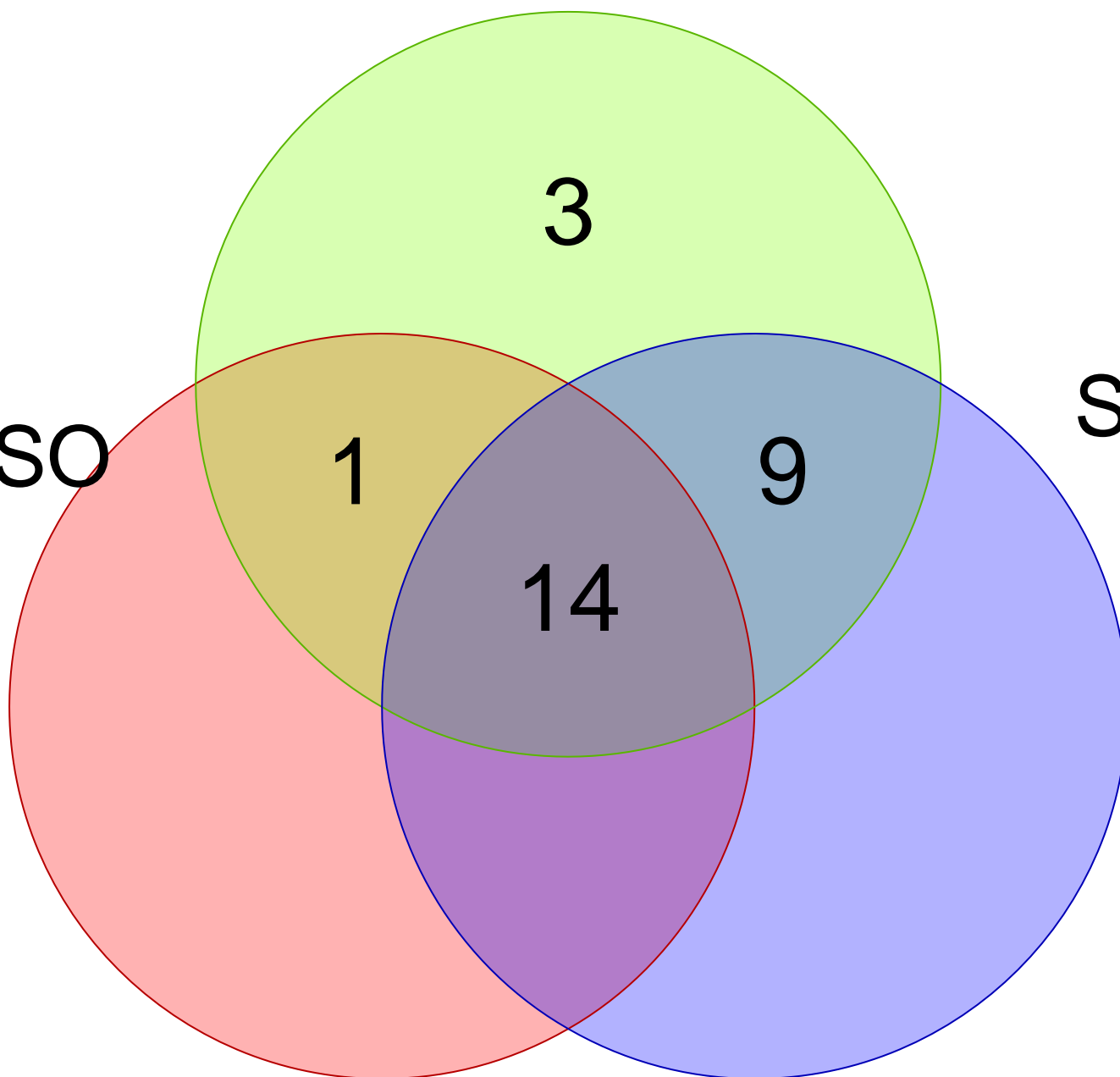

Supplement: S1 File — (ZIP) [file pone.0321751.s001.zip › 机器学习结果 27genes/Venny.pdf]

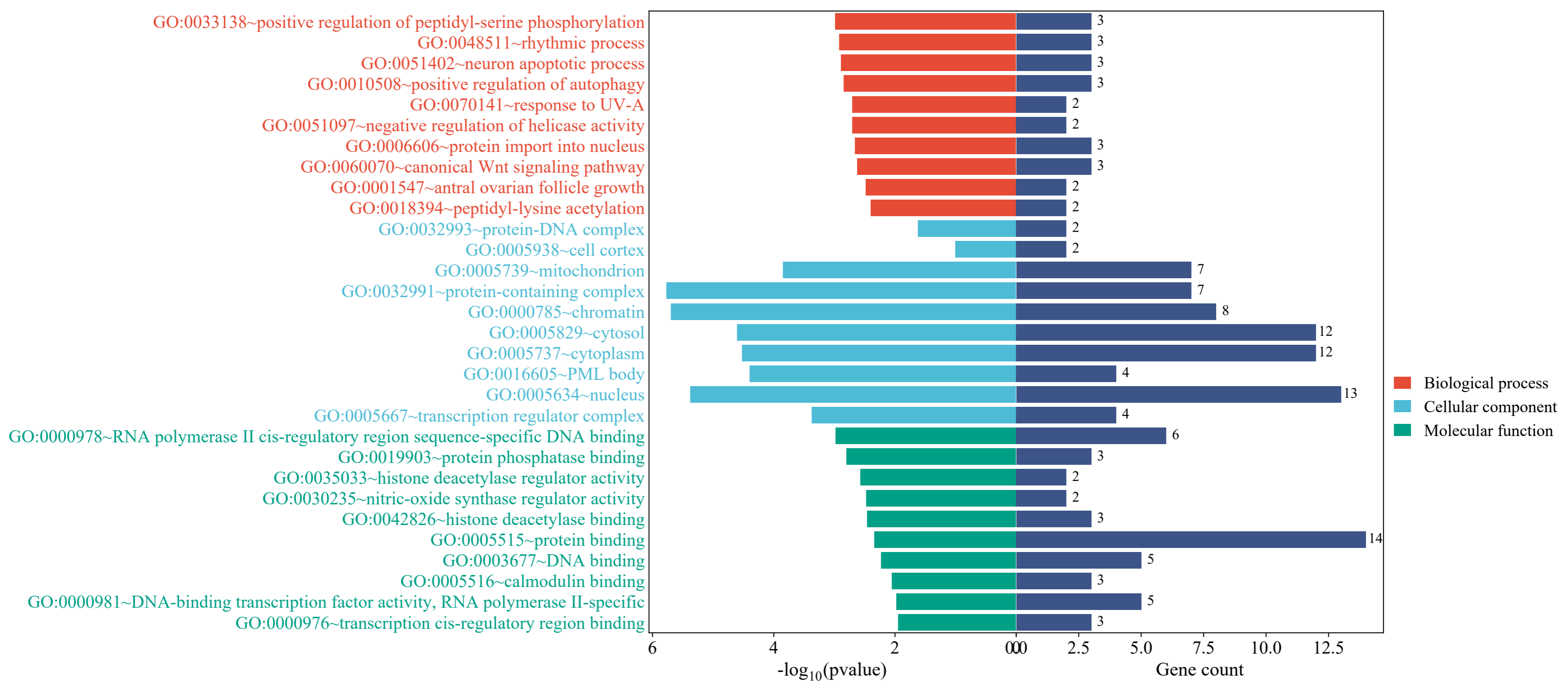

Supplement: S1 File — (ZIP) [file pone.0321751.s001.zip › GO_KEGG/GO.pdf]

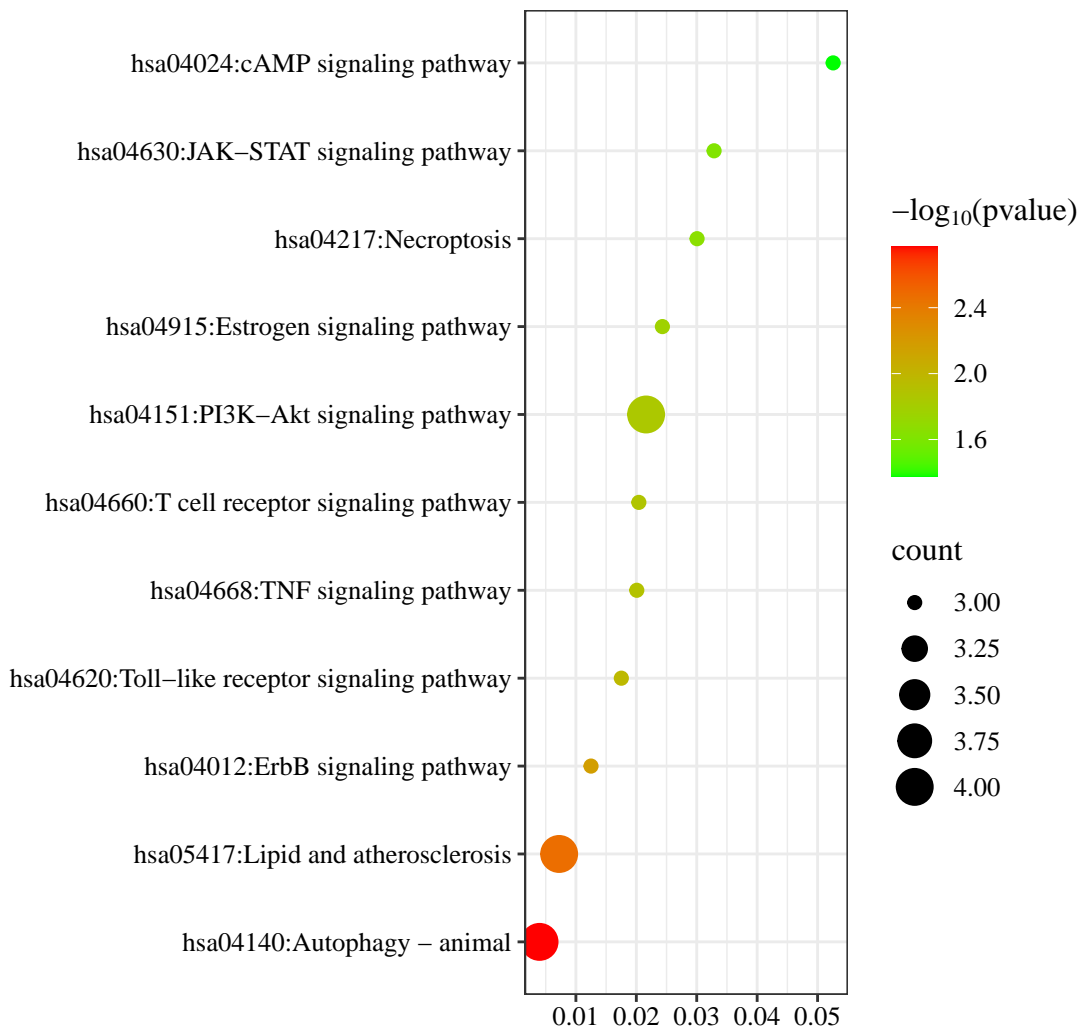

Supplement: S1 File — (ZIP) [file pone.0321751.s001.zip › GO_KEGG/KEGG.pdf]

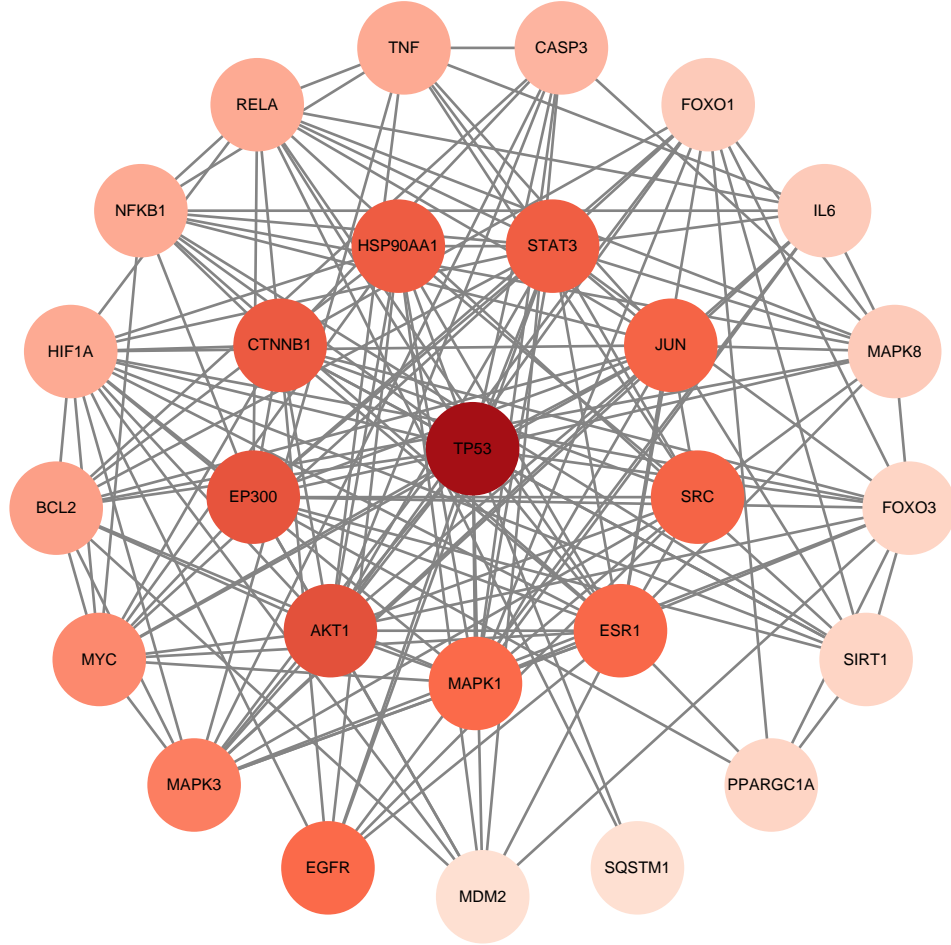

Supplement: S1 File — (ZIP) [file pone.0321751.s001.zip › PPI/图1-2 PPI.pdf]

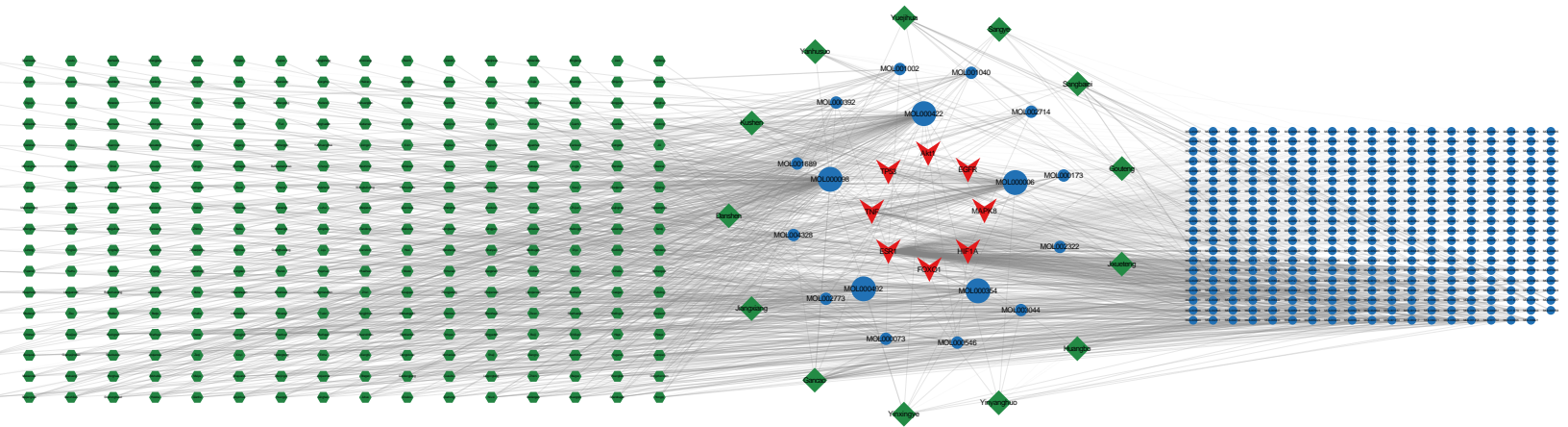

Supplement: S1 File — (ZIP) [file pone.0321751.s001.zip › 靶点_小分子_中药/中药-靶点-小分子可视化.pdf]

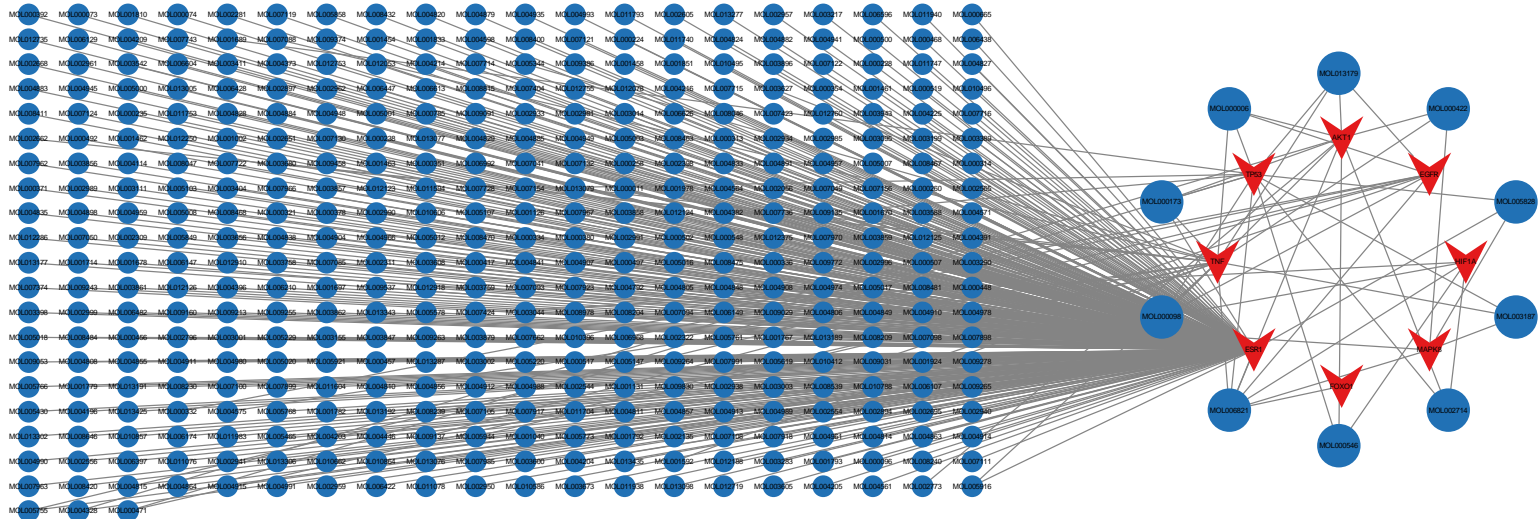

Supplement: S1 File — (ZIP) [file pone.0321751.s001.zip › 靶点对应小分子/靶点小分子.pdf]

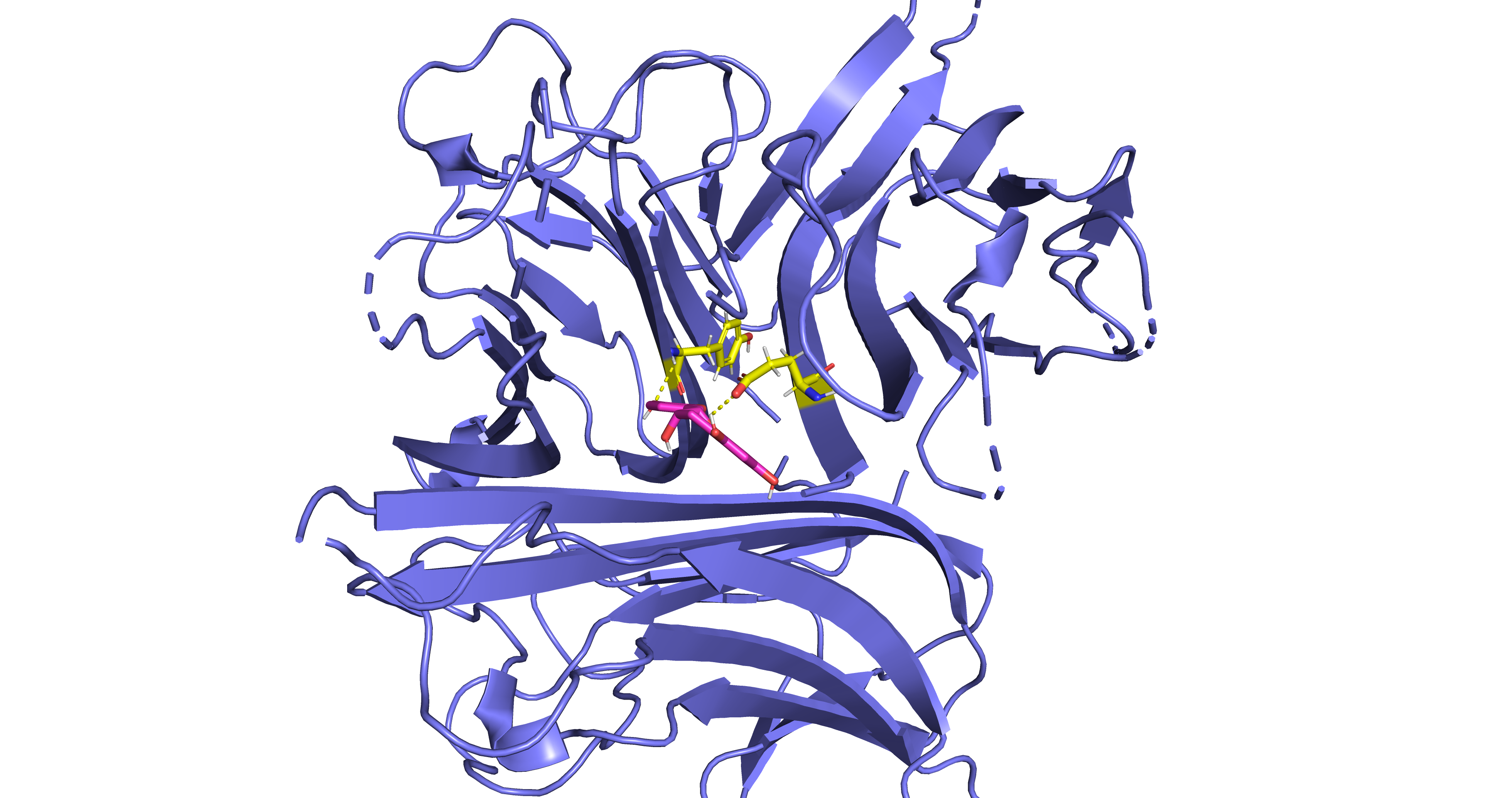

Supplement: S4 File — (ZIP) [file pone.0321751.s004.zip › 分子对接5/TNF/TNF-0073/input/TNF-0073-1.png]

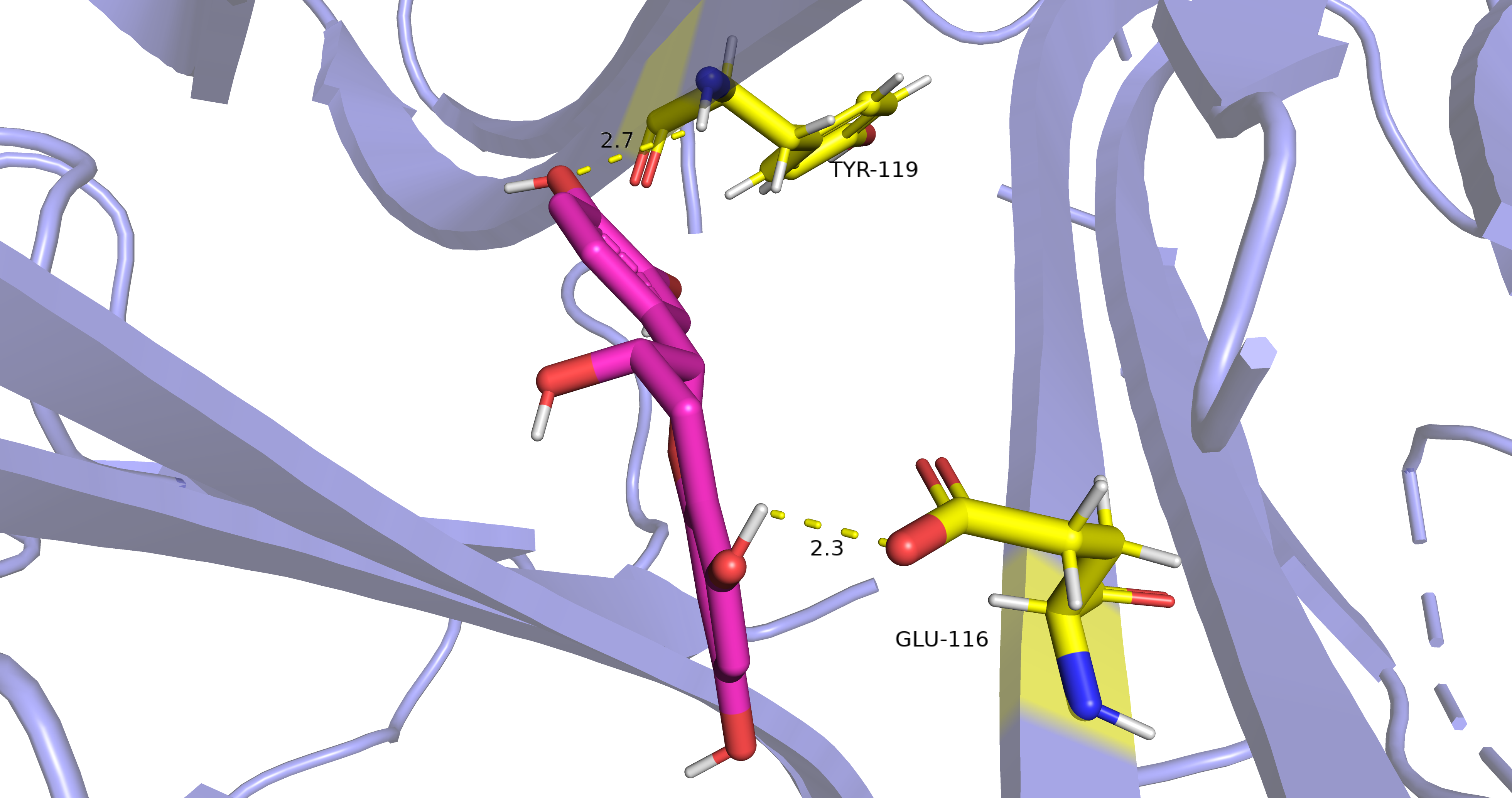

Supplement: S4 File — (ZIP) [file pone.0321751.s004.zip › 分子对接5/TNF/TNF-0073/input/TNF-0073-2.png]

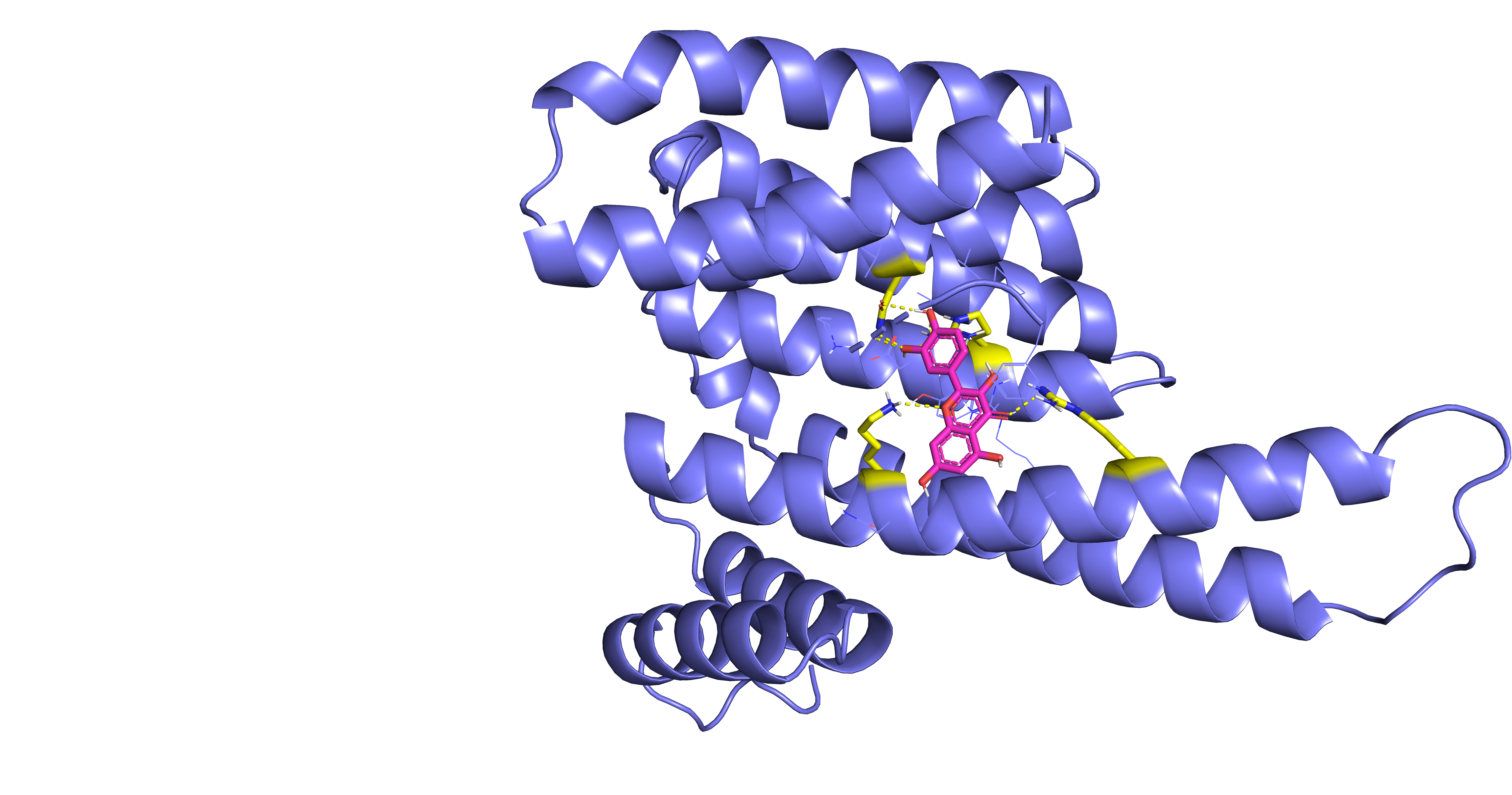

Supplement: S5 File — (ZIP) [file pone.0321751.s005.zip › 分子对接4/ESR1/ESR1-0098/results/ESR1-0098-1.png]

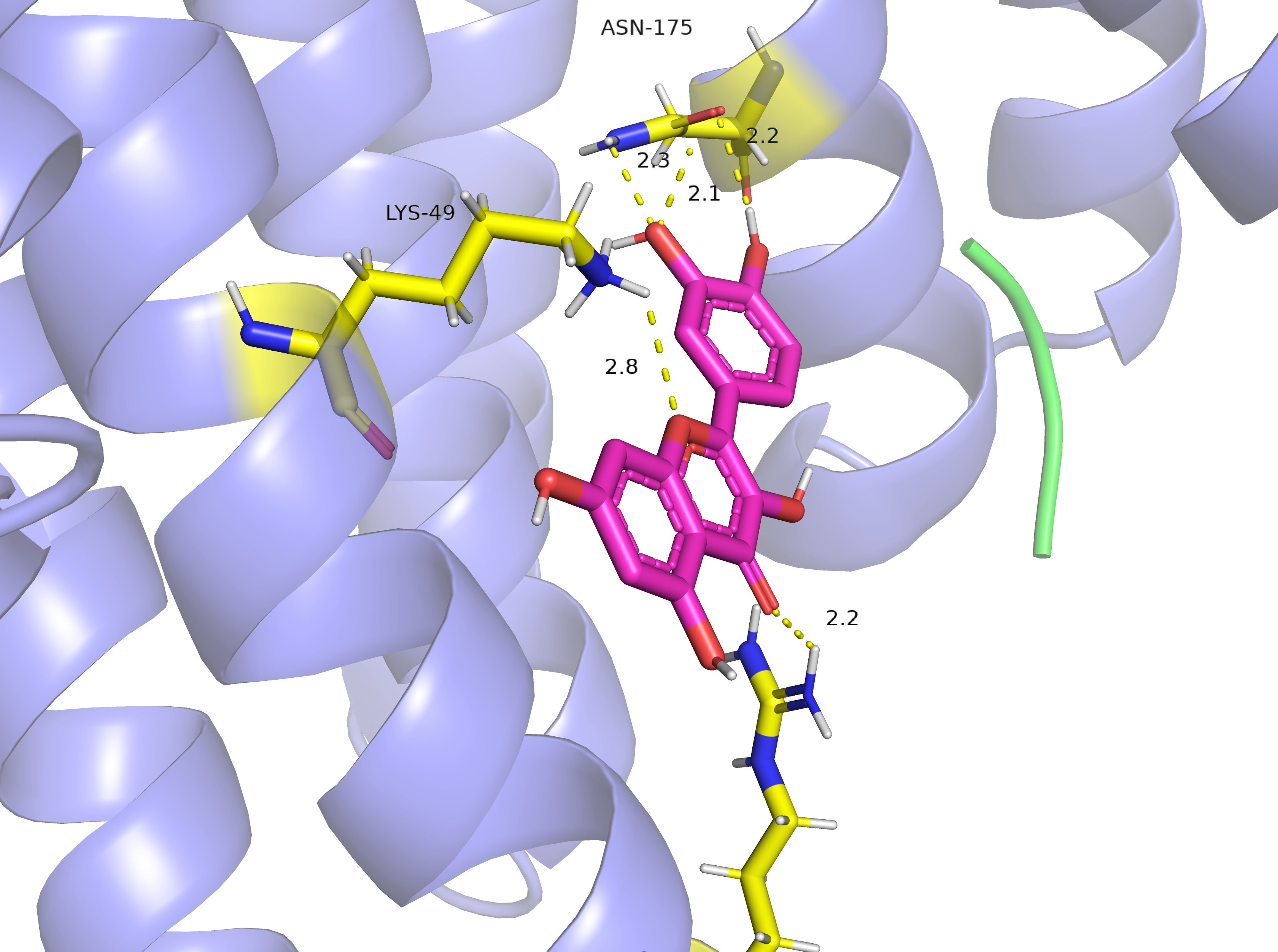

Supplement: S5 File — (ZIP) [file pone.0321751.s005.zip › 分子对接4/ESR1/ESR1-0098/results/ESR1-0098-2.png]

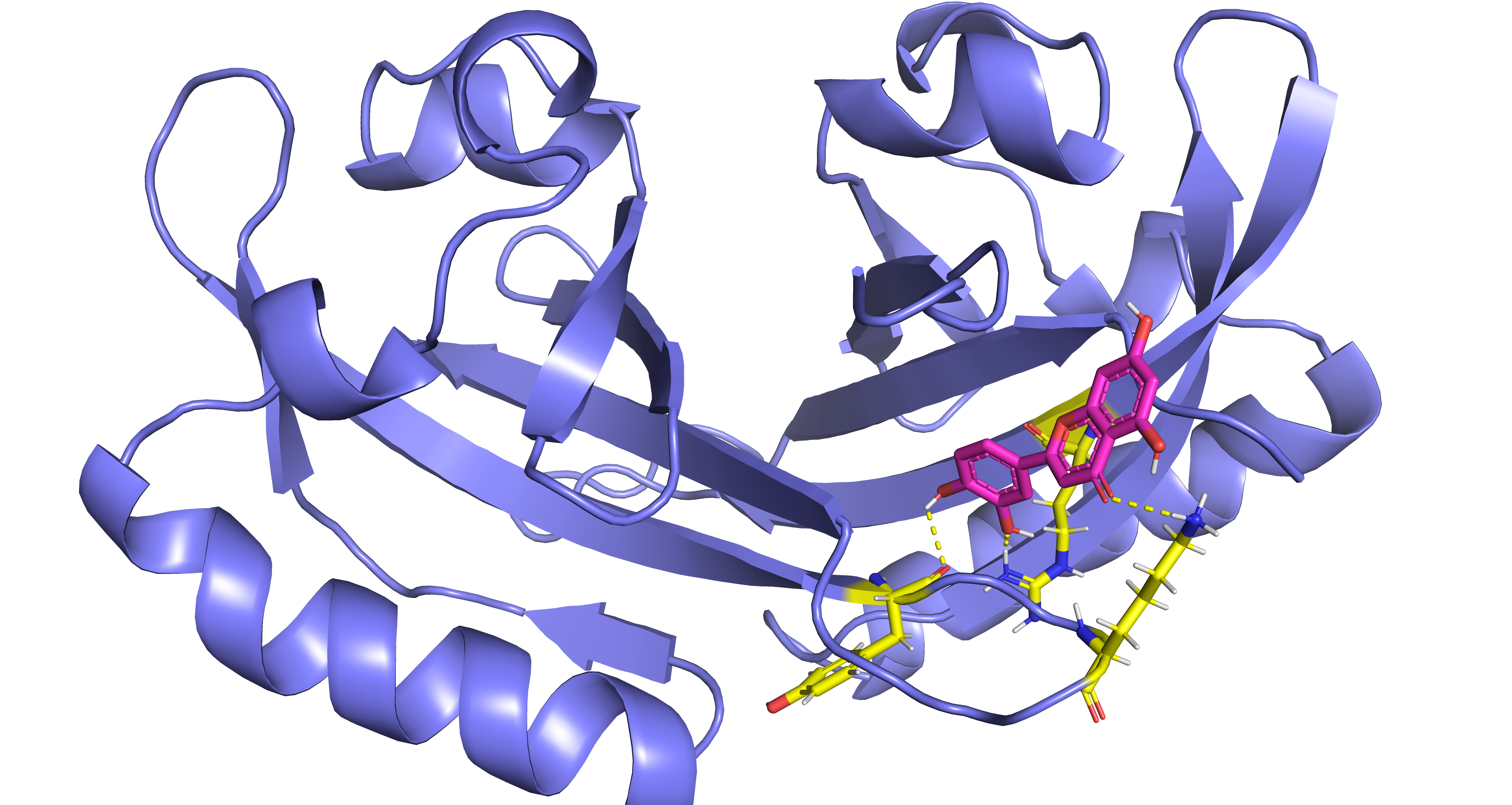

Supplement: S6 File — (ZIP) [file pone.0321751.s006.zip › 分子对接2/HIF1A/HIF-0006/input/HIF-0006-1.png]

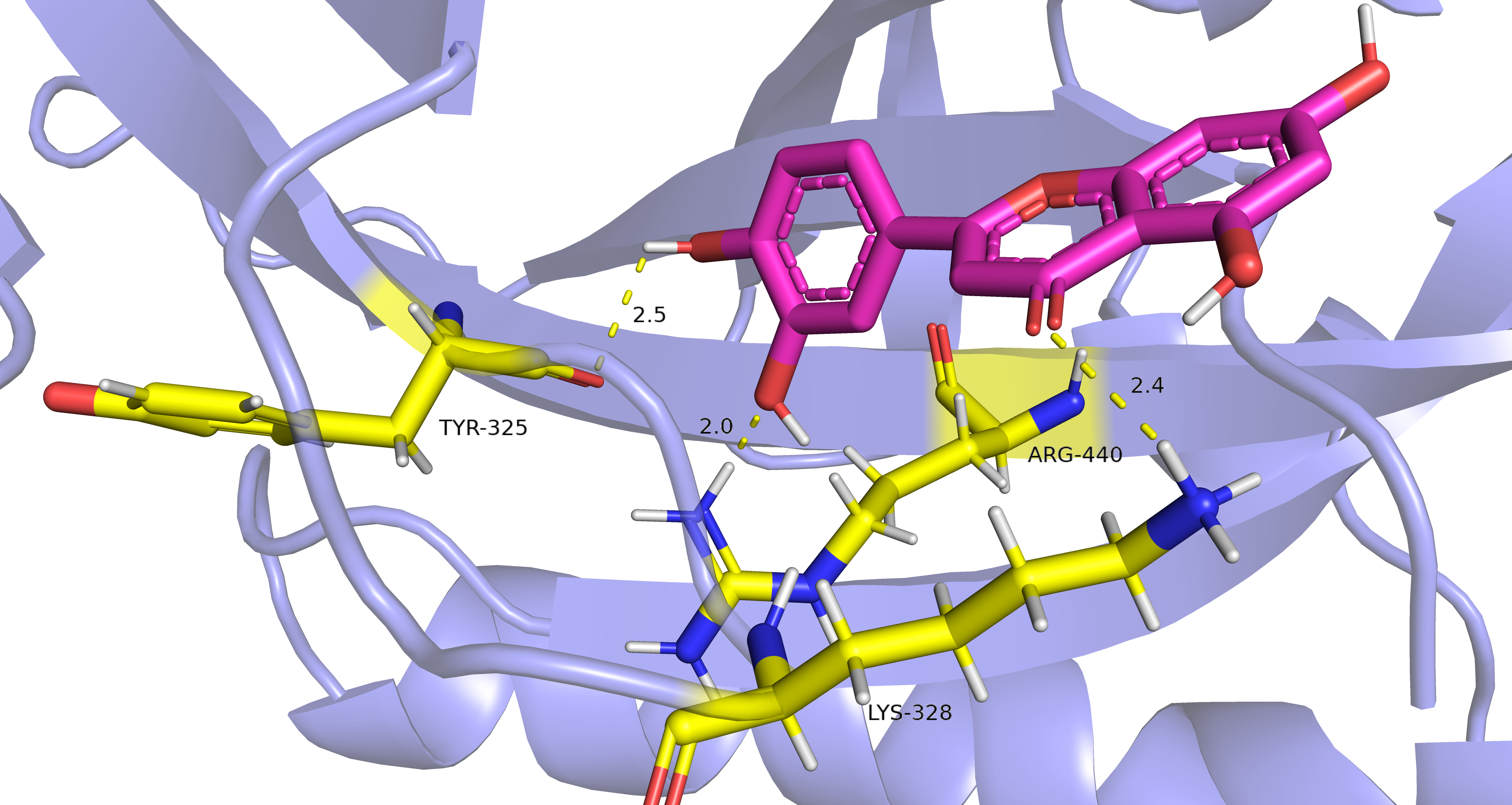

Supplement: S6 File — (ZIP) [file pone.0321751.s006.zip › 分子对接2/HIF1A/HIF-0006/input/HIF-0006-2.png]

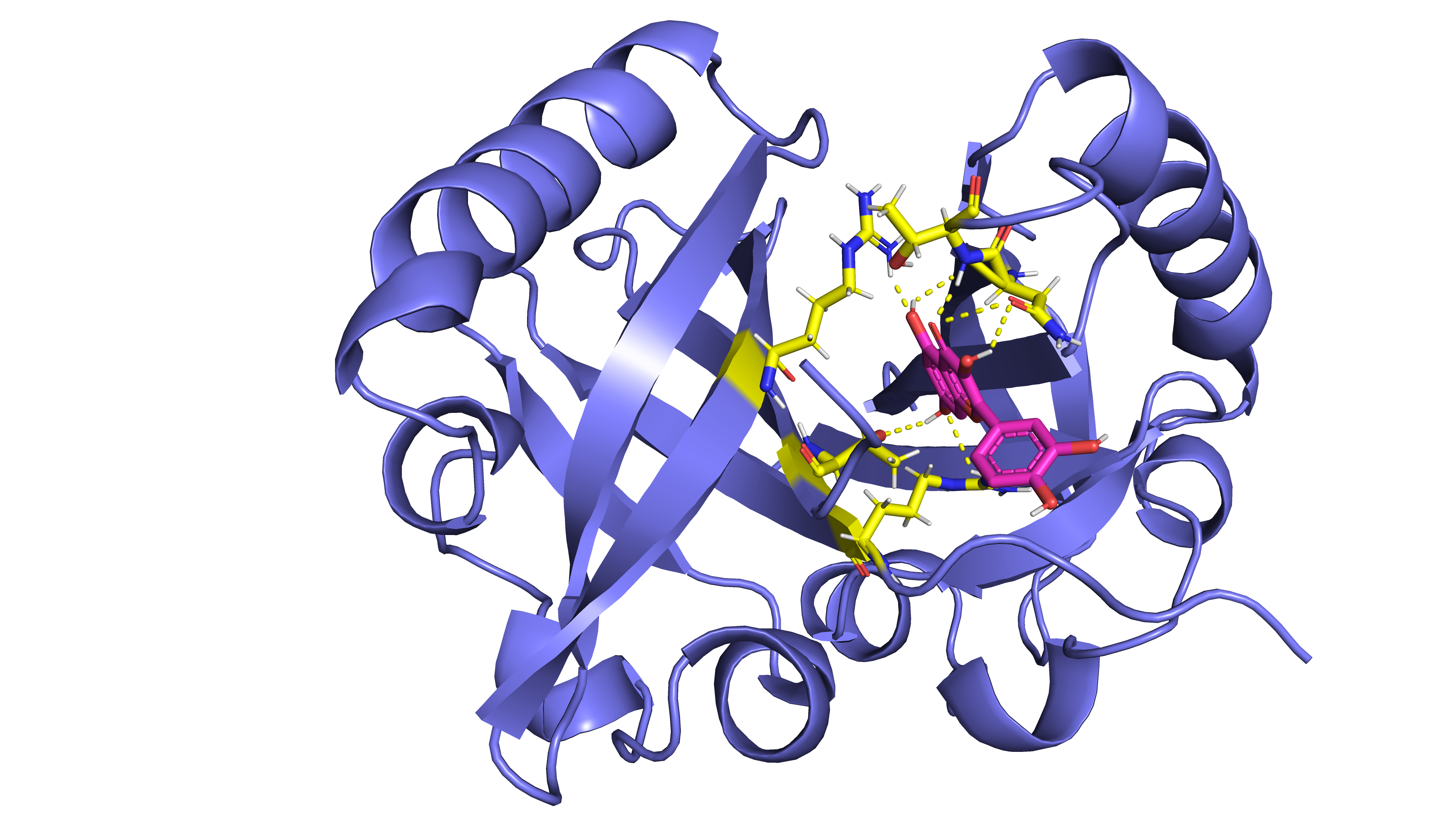

Supplement: S6 File — (ZIP) [file pone.0321751.s006.zip › 分子对接2/HIF1A/HIF-0098/input/HIF-0098-1.png]

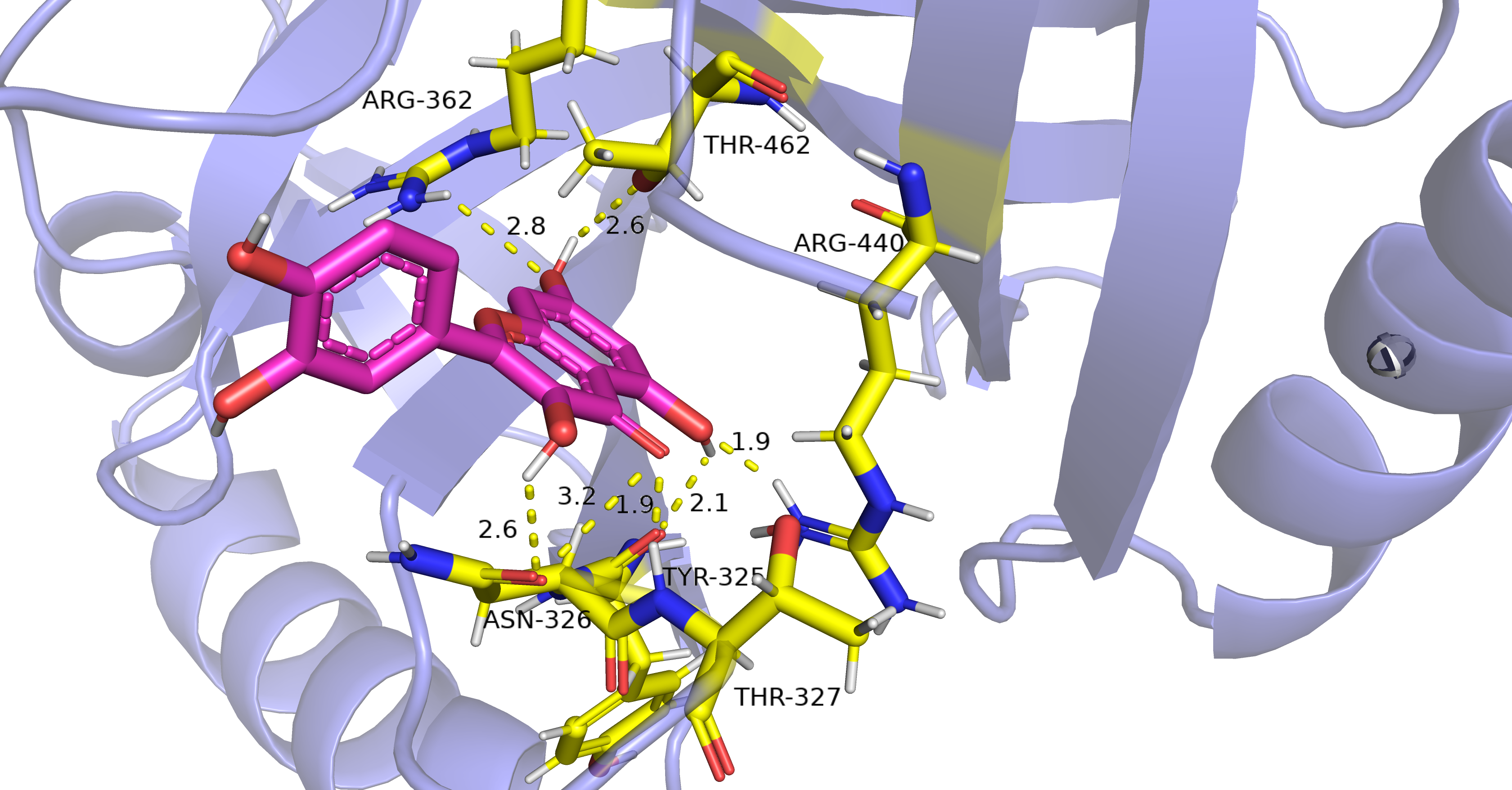

Supplement: S6 File — (ZIP) [file pone.0321751.s006.zip › 分子对接2/HIF1A/HIF-0098/input/HIF-0098-2.png]

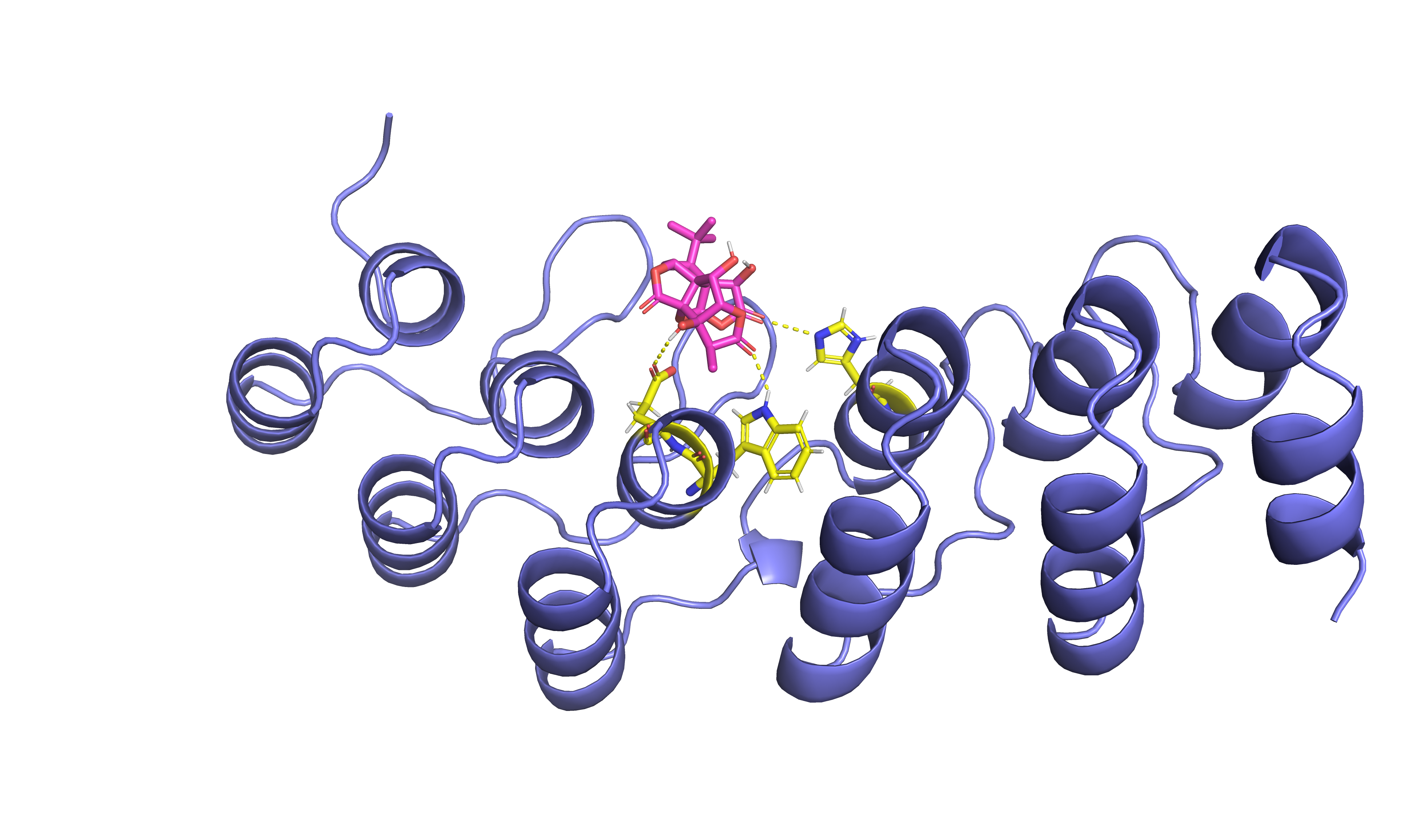

Supplement: S7 File — (ZIP) [file pone.0321751.s007.zip › 分子对接1/AKT1/AKT1-11061/input/AKT1-11061-1.png]

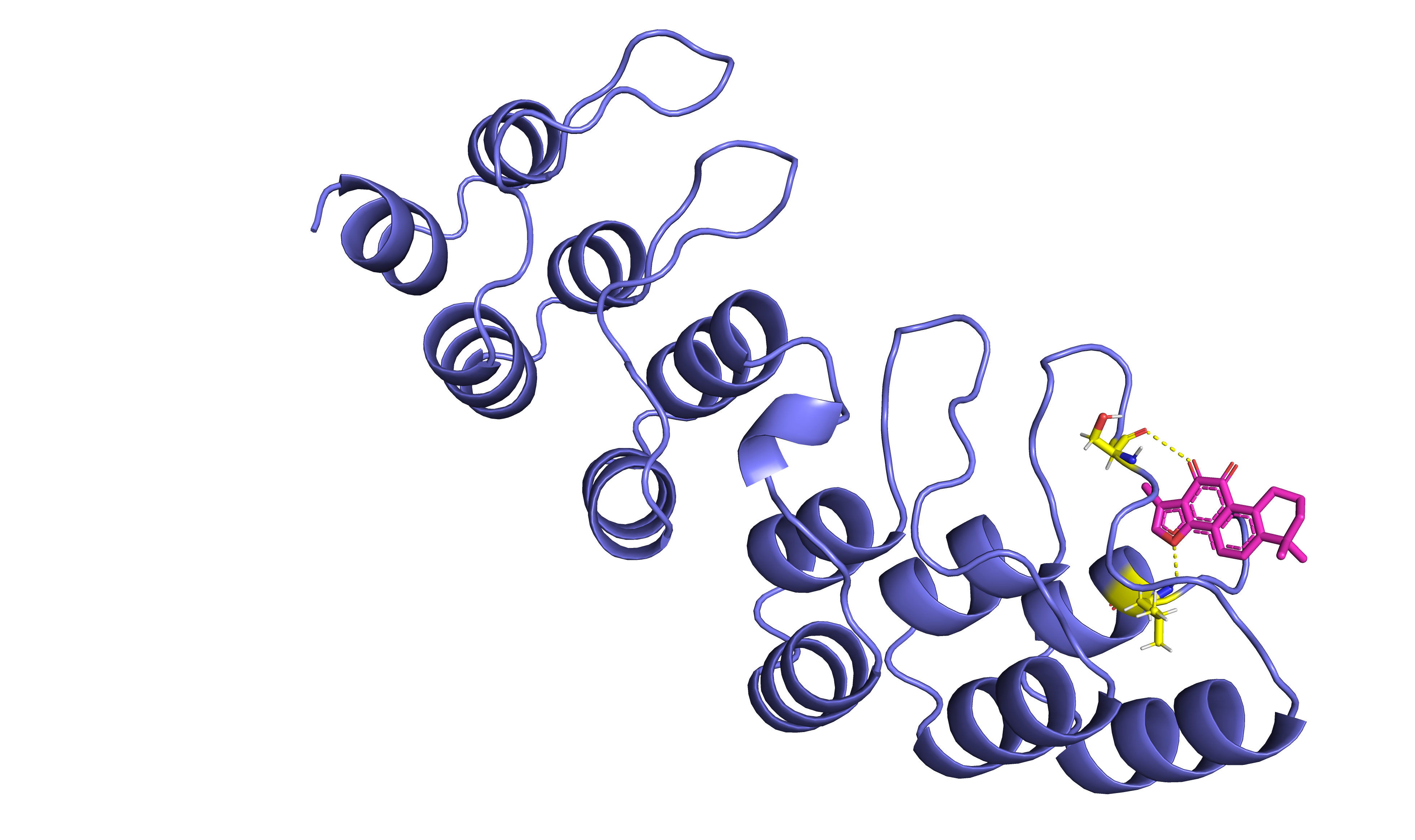

Supplement: S7 File — (ZIP) [file pone.0321751.s007.zip › 分子对接1/AKT1/AKT1-7154/results/AKT1_7154_1.png]

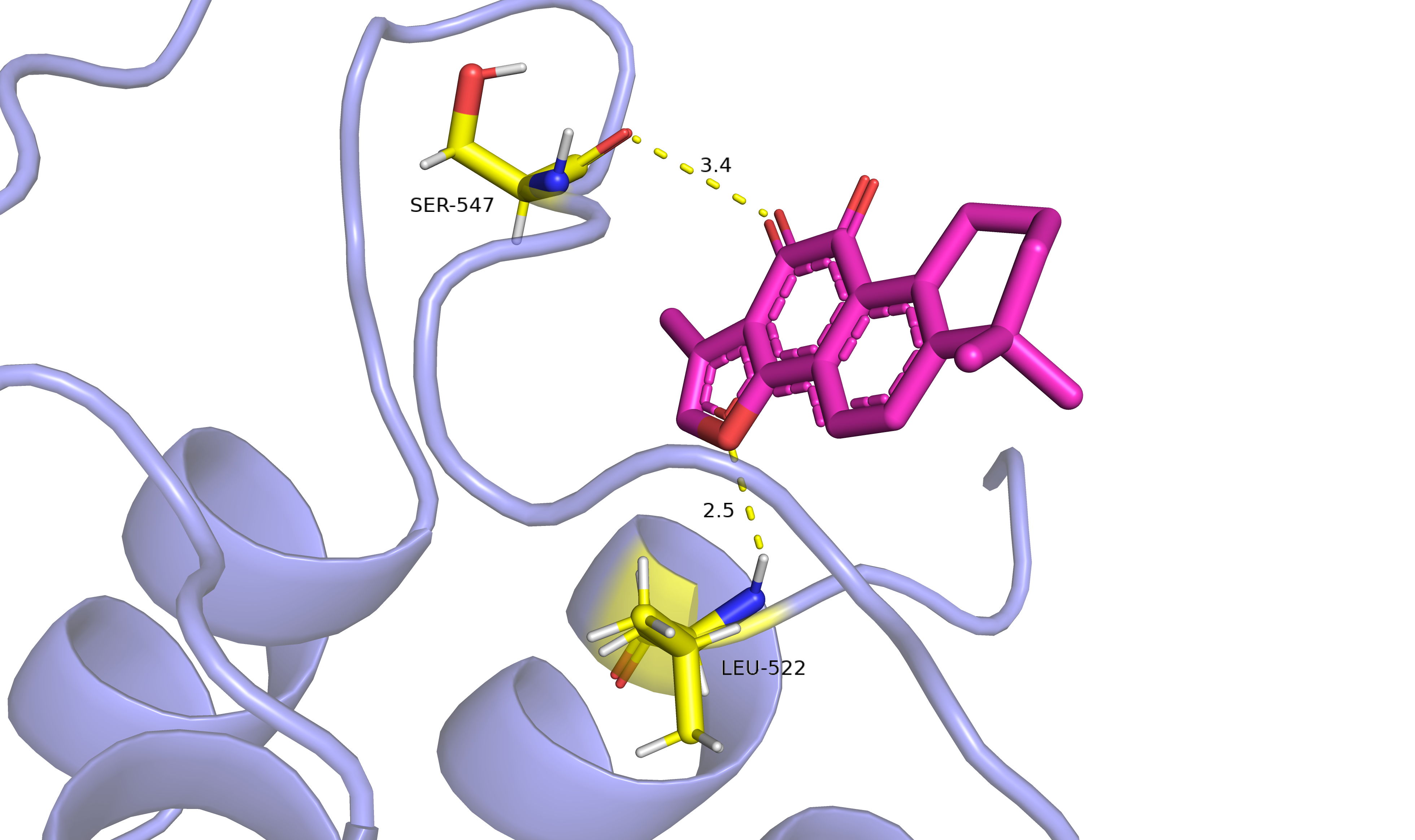

Supplement: S7 File — (ZIP) [file pone.0321751.s007.zip › 分子对接1/AKT1/AKT1-7154/results/AKT1_7154_2.png]
